# Secondary Metabolites and Biosynthetic Gene Clusters Analysis of Deep-Sea Hydrothermal Vent-Derived *Streptomyces* sp. SCSIO ZS0520

**DOI:** 10.3390/md20060393

**Published:** 2022-06-14

**Authors:** Huaran Zhang, Yingying Chen, Yanqing Li, Yongxiang Song, Junying Ma, Jianhua Ju

**Affiliations:** 1 Southern Marine Science and Engineering Guangdong Laboratory (Guangzhou), No.1119, Haibin Road, Nansha District, Guangzhou 511458, China; aifyui@126.com (H.Z.); chenyingying7788@163.com (Y.C.); liyanqing20@mails.ucas.ac.cn (Y.L.); songx@scsio.ac.cn (Y.S.); majunying@scsio.ac.cn (J.M.); 2 CAS Key Laboratory of Tropical Marine Bio-Resources and Ecology, Guangdong Key Laboratory of Marine Materia Medica, RNAM Center for Marine Microbiology, South China Sea Institute of Oceanology, Chinese Academy of Sciences, 164 West Xingang Road, Guangzhou 510301, China; 3 College of Oceanology, University of Chinese Academy of Sciences, Qingdao 266400, China

**Keywords:** polyketide, biosynthesis, *Streptomyces*, hydrothermal vent, deep sea

## Abstract

*Streptomyces* sp. SCSIO ZS0520 is a deep-sea hydrothermal vent-derived actinomycete. Our previous metabolism investigation showed that *Streptomyces* sp. SCSIO ZS0520 is a producer of cytotoxic actinopyrones. Here, another four types of secondary metabolites were identified, including six salinomycin isomers (**2**–**7**), the macrolide elaiophylin (**8**), the triterpene *N*-acetyl-aminobacteriohopanetriol (**9**), and the pyrone minipyrone (**10**). Among them, compounds **2**–**6** and **10** are new compounds. To understand the biosynthetic pathway of these compounds, a bioinformatic analysis of the whole genome was carried out, which identified 34 secondary metabolite biosynthetic gene clusters. Next, the biosynthetic pathways responsive to four types of products were deduced on the basis of gene function predictions and structure information. Taken together, these findings prove the metabolite potential of ZS0520 and lay the foundations to solve the remaining biosynthetic issues in four types of marine natural products.

## 1. Introduction

The hydrothermal vent environment is described as the most dynamic and extreme environment in the deep sea [1]. Because of the variable temperature and high level of toxic compounds or metals, the microorganisms that live there have evolved abilities to produce specific functional small molecules for adapting to the complicated habitat. Therefore, the microorganisms derived from extreme marine environments are exciting reservoirs of a variety of valuable drug candidates. *Streptomyces* sp. SCSIO ZS0520, the deep-sea hydrothermal vent-derived *Streptomyces*, was isolated from sediment in the Okinawa trough at a depth of −1039 m. In our previous metabolite exploration, a cytotoxic polyketide PM050511 and several new analogs were isolated from *Streptomyces* sp. SCSIO ZS0520, indicating its actinopyrone production ability [2]. Nevertheless, *Streptomyces* usually contains 20–40 biosynthetic gene clusters (BGCs), suggesting that the metabolic capacity of ZS0520 could be further explored.

Salinomycin (**1**), a clinically relevant antibiotic produced by *Streptomyces*, was first isolated in 1974 [3]. The crystal structure of this polyether antibiotic revealed the “head-to-tail” cyclized conformation by a hydrogen bond between the terminal carboxy group and the hydroxy group [4]. Because of the hydrophilic elements located in the interior region, salinomycin can ligate a Na^+^/K^+^ ion inside and carry it across biological membranes. The cation released inside cells destroyed the natural Na^+^/K^+^ ion gradients, ultimately leading to cell death [5]. Because of its ion-carrying ability, salinomycin exhibits a broad spectrum of pharmacological properties, including anticoccidiosis [6], antibacterial [7], antifungal [8], antiviral [8], and anticancer [9] activities. Previous studies have proved that salinomycin is susceptible to acidic, neutral, and alkaline environments, even at room temperature, which sometimes limits its usage and biosynthetic study [10,11,12]. Several degradation products of salinomycin have been identified, but the mechanism has not yet been thoroughly summarized [10,11,13]. Elaiophylins, also called efomycins, are symmetric macrolides dimerized from two identical polyketide monomers linked with two lactone bonds. These macrodiolides display remarkable biological properties, such as antibacterial, anticancer, and immunosuppressive activities [14]. *N*-acetyl-aminobacteriohopanetriol belongs to the bacteriophopane family. These triterpenes exist in many thermophilic bacteria, such as *Alicyclobacillus*, *Brevibacillus*, *Geobacillus*, *Meiothermus*, and *Thermus* [15]. Bacteriohopanetriols can insert themselves into the phospholipid bilayer to act as membrane reinforcers in prokaryotic systems, allowing adaption to extreme environments, such as low pH or high temperatures [16,17]. Pyrones are widely distributed in nature and display broad activities from signaling molecules to drug candidates [18]. In this paper, we report the isolation, structure elucidation, the whole genome sequence of ZS0520, and the biosynthetic pathway analysis of these secondary products.

## 2. Results and Discussion

### 2.1. Metabolite Investigation of the Strain Streptomyces sp. SCSIO ZS0520

ZS0520 mainly produces cytotoxic actinopyrones, as previously described [2]. By fermentation in a modified-RA medium, some other compounds were enriched. The culture was extracted with butanone, and mycelia were extracted with acetone. The extracts were combined and then subjected to silica gel column chromatography (CC), reversed-phase medium-pressure preparative liquid chromatography (RP-MPLC), Sephadex LH-20 CC, and semipreparative HPLC to yield nine natural products (NPs), including six new compounds: *seco*-salinomycins A–E (**2**–**6**) and minipyrone (**10**).

*Seco*-salinomycins A and B (**2** and **3**) were isolated as pale yellow amorphous powders. By analyzing the HRESIMS spectrum (*m*/*z* 771.4644 [M + Na]^+^, calcd. for 771.4654), their molecular formula was established to be C_42_H_68_O_11_. The molecular formulae and the NMR assignments were comparable to those of salinomycin (**1**), a well-known veterinary medicine (Table S1) [10]. However, the ^1^H and ^13^C NMR spectra showed two sets of similar signals at a 1:1 ratio. Two pairs of olefinic, three carbonyls, and several oxygen-linked carbons were found in each group of signals. Most signals matched the NMR of salinomycin except for the C-16–C-21 region. The HMBC correlations from H-18 to C-16, C-17, C-19, and C-20 and H-19 to C-17 and C-20 showed the presence of conjugated double bonds at C-16/C-17 and C-18/C-19 and a carbonyl group at C-20 (Figure S1). The existence of the oxygenated olefinic C-17 and HMBC correlation from H-13 to C-17 indicated an opened C-17–C-21 ring, different from salinomycin (**1**). The hemiacetal moiety at C-21 can spontaneously change to a hydroxy and a carbonyl group and reclose again to form epimers of **2** and **3**. This was also demonstrated by the relative differing chemical shifts at C-21–C-24 due to the shielding effect of the OH-21. On the basis of the above data, the structures of **2** and **3** were elucidated as a pair of epimers at C-21 and named *seco*-salinomycins A and B.

*Seco*-salinomycin C (**4**) was isolated as a pale yellow amorphous powder. The HRESIMS spectrum showed the molecular formula was C_29_H_46_O_7_ (*m*/*z* 529.3135 [M + Na]^+^, calcd. for 529.3136), corresponding to a loss of C_13_H_22_O_4_ compared with **2** and **3**. Its ^1^H and ^13^C NMR spectra were similar to those of compounds **2** and **3**, except that the signals corresponding to C-21–C-33 were missing. The low-field carbon signal δ194.9 with its proton signal of δ9.56 d (*J* = 8.4) indicated the existence of an aldehyde group. With the help of the remaining 2D NMR, the structure of **4** was identified as a salinomycin missing C-21–C-33 and named *seco*-salinomycin C.

*Seco*-salinomycins D and E (**5** and **6**) were isolated as pale yellow amorphous powders. The HRESIMS spectra of **5** and **6** exhibited the same pseudo-molecular ion peak at *m*/*z* 751.4970 [M − H]^−^ (calcd. for 751.4996), suggesting the molecular formula of C_42_H_72_O_11_. Compounds **5** and **6** also exhibited a pair of NMR signals at a ratio of 1:1, implying the mixture of two isomers (Table S2). The ^1^H NMR and ^13^C NMR spectra of **5** and **6** comprised resonances similar to those of salinomycin except for C-16–C-21 [10]. The COSY signals for H-18–H-19–H-20, together with the HMBC correlations from H-18 to C-17 and H-20 to C-21 and C-22 indicated an *α*,*β*,*γ*,*δ*-unsaturated carboxylic chain. The terminal carboxylic ester was fused at C-13 according to the analysis of HMBC correlation from H-13 to C-17 and the evidence of an increased chemical shift of C-13 (δ75.8). The absence of HMBC correlations from H-15, H-16, or H-34 to C-17, together with the evidence of methylene-type C-16, confirmed the 16,17-*seco*-B ring. The chemical shifts and peak shapes of H-20 protons in two sets of signals were significantly different, with 5.58, dt (12.2, 1.6), and 5.16, d (11.6) being the major differences between **5** and **6**. The ^4^*J* W-type long-range coupling by H-22 caused the dt-shaped signal of H-20 with a 1.6 Hz coupling constant in **6**. The d-shaped H-20 resonance of **5** was located opposite to H-22’s W position, indicating the *Z*- or *E*-positions at the C-20/C-21 double bond. On the basis of the above data, the structures of **5** and **6** were elucidated and named *seco*-salinomycins D and E. As for the absolute configuration of these *seco*-salinomycins, the biosynthetic gene cluster *sln* showed 100% similarity with the reported gene sequence, indicating that the stereochemistry is completely the same as that of the reported structure [4]. Since no additional chiral carbon was formed during the degradation process, we maintained the previously reported absolute conformation.

Minipyrone (**10**) was isolated as a yellowish oil. The molecular formula was established as C_11_H_16_O_3_ by HRESIMS ([M + H]^+^ found at *m*/*z* 197.1173, calcd. for 197.1172). Two pairs of olefinic signals with a carboxyl carbon at a higher field indicated a pyrone moiety. According to the strong HMBC correlation from H-11 to C-3, the methoxy group was bound at C-3. The linkage of the isopentyl at C-1 was demonstrated by HMBC correlations from H-7 to C-1. With the aid of the remaining information, the structure of **10** was identified. Since compound **10** was smaller than the actinopyrones previously isolated from this strain, we named it minipyrone.

**Figure 1:**
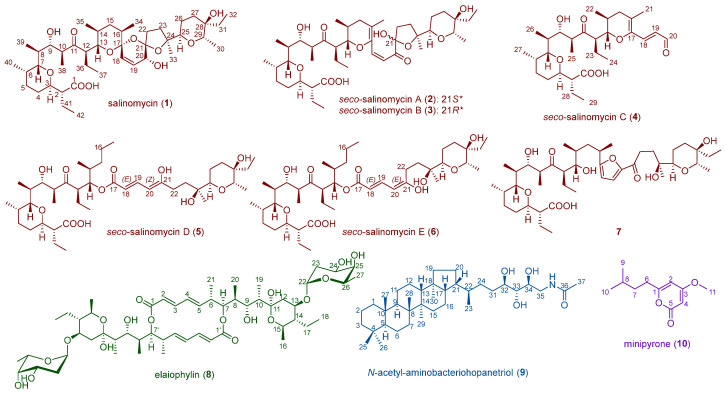
Structures of compounds isolated from *Streptomyces* sp. SCSIO ZS0520 (* the absolute configuration of compounds **2** and **3** are interchangeable).

Compounds **7**–**9** were elucidated by comparing their spectral data with those reported in the literature [10,19,20]. They were identified as an unnamed isomer of salinomycin (**7**), elaiophylin (**8**), and *N*-acetyl-aminobacteriohopanetriol (**9**), respectively. The structures of these compounds are given in Figure 1.

### 2.2. Genome Features and Annotations of Strain Streptomyces sp. SCSIO ZS0520

**Figure 2:**
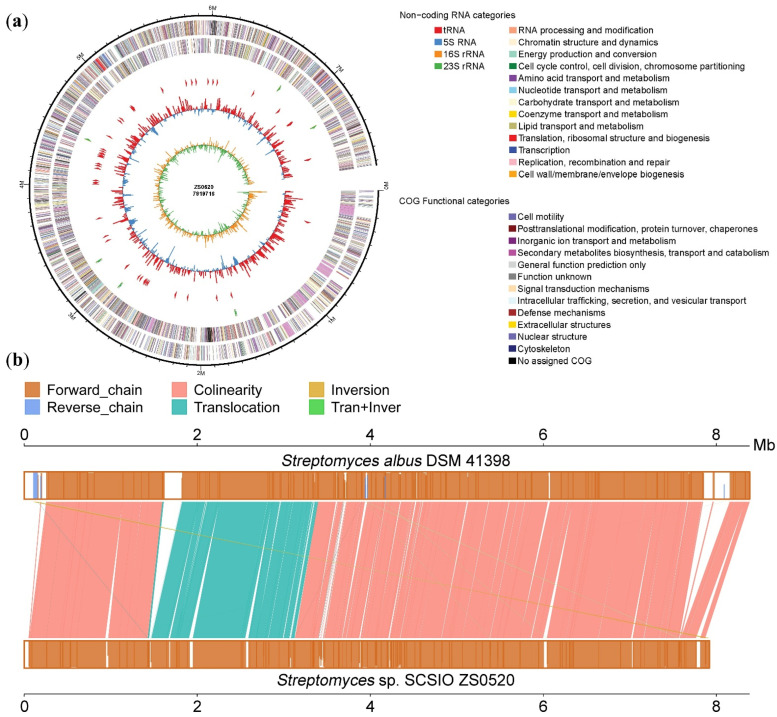
(**a**) The complete genomic graph of *Streptomyces* sp. SCSIO ZS0520. The six circles (from outer to inner) represent the size of the genome, CDs on positive chains, CDs on negative chains (different colors represent the functional classification of cogs with different CDs), rRNA and tRNA, GC content, and GC skew value; (**b**) the comparative genomics graph between *Streptomyces* sp. SCSIO ZS0520 and *Streptomyces albus* DSM 41398.

With an Illumina and PacBio hybrid strategy, the high-quality genome sequence of ZS0520 was carried out and revealed a 7,919,716 bp linear chromosome with an average G + C content of 72.85%. The complete genome was predicted to contain 5956 coding DNA sequences (CDSs), 65 tRNA genes, and 18 rRNA genes (Table 1, Figure 2a). On the basis of the genome sequence, we carried out the taxonomic classification of ZS0520 by the maximum Average Amino Acid Identity (AAI) found against all the genomes in the database using the Microbial Genomes Atlas (MiGA) website. The sequence matched the genome of the genus *Streptomyces*, indicating that ZS0520 is a member of *Streptomyces* (Figure S2). In a comparison of the functional annotation of the predicted genes, ZS0520 shared 5481 homologous genes with *Streptomyces albus* DSM 41398 (accession number: PRJEB11297), accounting for 92% of CDSs in ZS0520 [21]. In Figure 2b, the upper box indicates the *Streptomyces albus* DSM 41398 genome, and the lower box is *Streptomyces* sp. SCSIO ZS0520 sequence. The heights of the lines in the two boxes indicate the similarity of the genes in the two strains, from 0% to 100%. Almost all homologous genes had >90% similarity in colinear, translocation, inversion, and translocation and inversion modes. The results indicate that ZS0520 and DM41398 are genetically related.

To identify the secondary metabolite BGCs of ZS0520, the whole genome sequence was uploaded to antiSMASH tools. The genome encoded at least 34 putative BGCs, including nine polyketide BGCs (Type I or Type II PKS); five nonribosomal peptide clusters (NRPS); five terpene clusters; and several other BGCs, such as lanthipeptide, siderophore, butyrolactone, phenazine, and so on (Table S3). This finding suggests that ZS0520 possesses the potential to produce at least 34 different types of NPs with distinct skeletons. Cluster 10 (*atpn*), responsible for actinopyrone biosynthesis, was proposed in our previous study [2]. Clusters 3, 32, and 34, related to salinomycin, *N*-acetyl-aminobacteriohopanetriol, and elaiophylin, were identified by comparisons with the literature [19,22,23]. Clusters 14, 16, 18, 25, 30, and 31 showed 100% identities with known BGCs for producing albaflavenone, albusnodin, melanin, SAL-2242, melanin, and ectoine, indicating the potential biosynthetic ability of ZS0520. The remaining 24 clusters had low similarity with previously reported BGCs and might be potential sources for genome mining.

### 2.3. Biosynthesis and Isomeric Mechanism of Salinomycin

**Figure 3:**
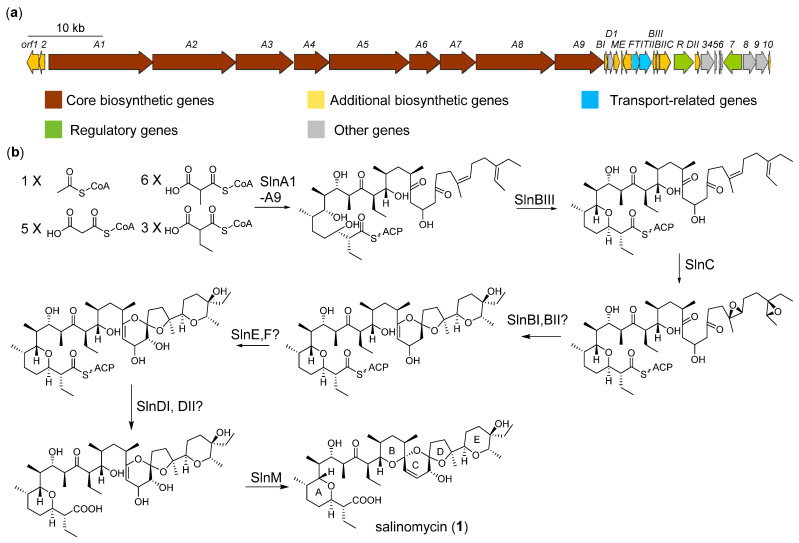
Proposed biosynthetic gene cluster (**a**) and pathway (**b**) of salinomycin.

The *sln* cluster (cluster 3 in the ZS0520 genome) was highly homologous to the salinomycin cluster from *Streptomyces albus* [21,22,24]. As previously reported, the polyketide chain is formed by nine core PKS (SlnA1–A9) using one acetyl-CoA and extends with five malonyl-CoA, six methylmalonyl-CoA, and three ethylmalonyl-CoA units (Figure 3, Table S4). Before being released from ACP, the epoxide hydrolase SlnBIII catalyzes the A ring formation, which is followed by oxidization by the epoxidase SlnC to form a diepoxide at C-24/C-25 and C-28/C-29 [25]. The other two epoxide hydrolases, SlnBI and SlnBII, are presumed to open the epoxy ring in tandem to cyclize the C, D, and E rings. The hydroxylation at C-20 is proposed to be catalyzed by SlnE and or SlnF. The thioesterases SlnDI and or SlnDII are proposed to release the polyether chain from PKS. Finally, the methyltransferase-like enzyme SlnM catalyzes the spirocyclization-coupled dehydration of OH-19, leading to the final cyclization of the B ring [22].

We proposed that *seco*-salinomycins A–E (**2**–**6**) and **7** are degraded from ionophore antibiotics salinomycin, which is susceptible to protic solutions at room temperature. Therefore, we analyzed the fresh extract from ZS0520. The mass corresponding to the absorption peak at *t*_R_ 24.5 min was identified as C_32_H_48_O_9_ ([M + H]^+^ *m*/*z* 751.5014, calcd. for 751.4991), which is consistent with the molecular formula of salinomycin (Figure S36). No obvious peaks corresponding to **2**–**7** were discovered in the crude extract. This proved bona fide product in ZS0520 was salinomycin, and **2**–**7** were degraded from the salinomycin. Several degradation products of salinomycin and its analog, narasin, have been identified in previous research [10,11,13]. By summarizing the features of these degradation products, we divided the degradation processes into four routes (Figure S3). Route 1 starts with the protonation of the oxygen in the C ring, forming a hemiacetal structure at C-21 and a double bond at C-16/C-17. Since the hemiacetal group is unstable, the continuous interconversion results in the formation of two epimers, *seco*-salinomycins A and B (**2** and **3**), with different configurations at C-21. In contrast to the expected product of a ring opening, compounds 2 and 3 contain a carbonyl group at the C-20. Whether the oxidation process is spontaneous or enzyme-catalyzed is unclear. Route 2 is similar to route 1 but leads to the cleavage of the C-20/C-21 bond. The intermediate can undergo spontaneous dehydrogenation and rearrangement to form *seco*-salinomycin C (**4**), or OH-20 attacks C-17 to yield another unnamed product [11]. The degradation mechanism of compound **7** operates through the protonation-induced rearrangement of the B, C, and D rings [10]. Compounds **5** and **6** are proposed to undergo route 4. The dehydration of OH-20 induces the breakage of the C-16/C-17 bond. The D ring is then hydrolyzed into two OH groups, leaving C-21 as an unstable hemiacetal moiety. The newly formed C ring rearranges again to form *seco*-salinomycin D or E (**5** and **6**). Because of the *keto-enol* tautomerism, *seco*-salinomycins D and E can interconvert with each other to yield different configurations at the C-20/C-21 double bond.

### 2.4. Proposed Biosynthetic Pathway of Elaiophilin

**Figure 4:**
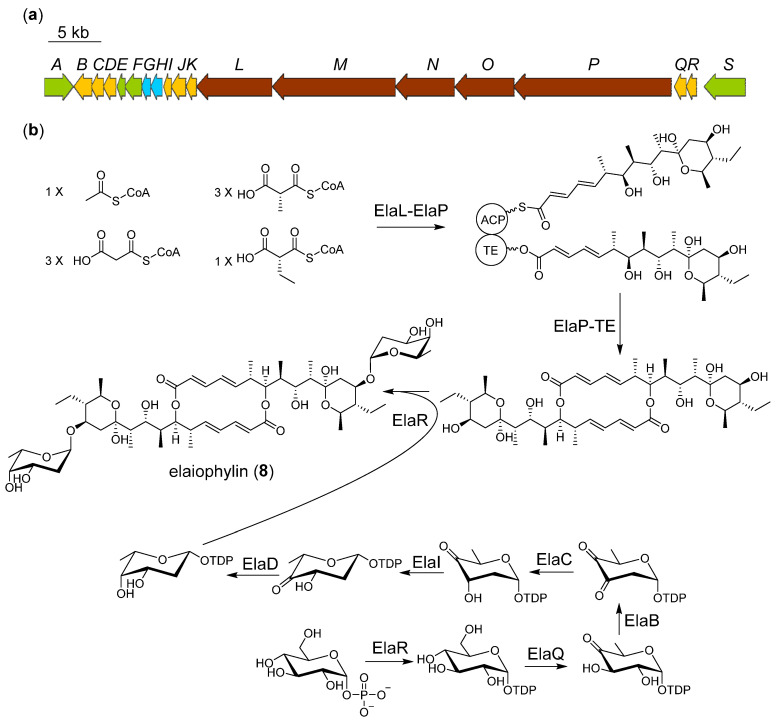
Proposed biosynthetic gene cluster (**a**) and pathway (**b**) of elaiophilin.

Elaiophylin (**8**) is a two-glycosyl substituted macrodiolide consisting of a symmetrical 16-membered decaketide diolide skeleton with two 2-deoxy-l-fucose moieties. The biosynthetic gene cluster of elaiophylin has been identified in species of *Streptomyces* [14]. One of the most interesting aspects of elaiophylin biosynthesis is the reuse of the TE domain, which catalyzes condensation twice between the TE-bound and the ACP-bound monomers to form the macrodiolide skeleton [19]. We presume that the formation of two pyran rings is likely to be spontaneous. As mentioned in the previous salinomycin, the carbonyl and hydroxyl groups can interchange into a hemiacetal structure. In the separation process, we also observed that the three absorption peaks with different retention times all turned into elaiophilin after purification. The substituent 2-deoxy-l-fucose was also discovered in brasilinolide, aclacinomycin, marcellomycin, and esperamicin [26]. The genes *ElaB*, *C*, *I*, *D* located in this cluster are homologous to the *nbrC*, *D*, *E*, *F* harbored in the brasilinolide biosynthetic gene cluster, which may be associated with 2-deoxy-l-fucose biosynthesis (Figure 4, Table S5) [26]. It can be proposed that the biosynthesis of the glycosyl in elaiophylin begins with glucose phosphorylation; this phosphatase is usually located outside the gene cluster. Then, the glucose-1-phosphate thymidylyltransferase (ElaR) changes the glucose-1-phosphate to its dTDP form. The dTDP-glucose 4,6-dehydratase (ElaQ), dTDP-4-keto-6-deoxy-hexose 2,3-dehydratase (ElaB), dTDP-4-keto-6-deoxy-hexose 2,3-reductase (ElaC), dTDP-4-keto-6-deoxy-hexose 3,5-epimerase (ElaI), and dTDP-4-keto-6-deoxy-hexose 4-ketoreductase (ElaD) tailor the dTDP-glucose step by step. The glycosyltransferase ElaJ finally fuses the dTDP-2-deoxy-l-fucose to the two terminal hydroxyl groups of decaketide diolide skeleton to yield elaiophylin (**8**). Some methylation derivatives on unsubstituted hydroxyl were identified previously; growing evidence suggests that these derivatives are artifacts of the use of methanol as a solvent [27].

### 2.5. Proposed Biosynthetic Pathway of N-Acetyl-aminobacteriohopanetriol

**Figure 5:**
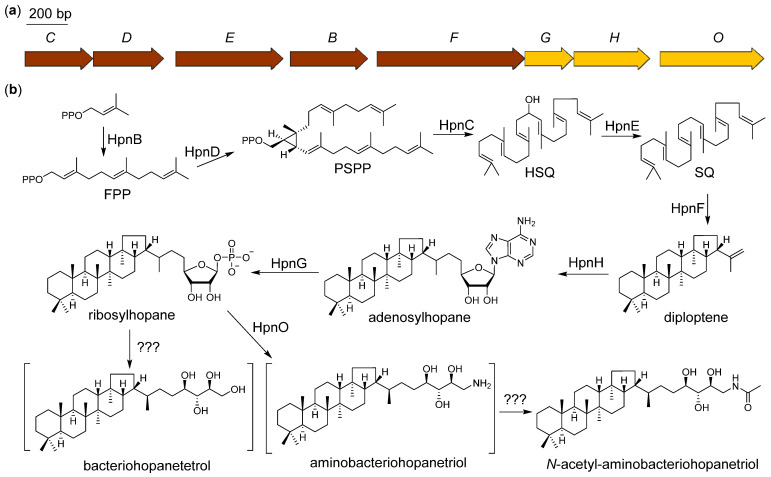
Proposed biosynthetic gene cluster (**a**) and pathway (**b**) of *N*-acetyl-aminobacteriohopanetriol.

Bacteriohopanepolyols are pentacyclic triterpenoids, which mainly occur in prokaryotes and promote the permeability and stability of the cell membrane [17]. All pentacyclic triterpenoids are synthesized using six isopentenyl units. Although eukaryotes and prokaryotes share the same precursors and final skeleton, the biosynthetic pathways are different, from the isopentenyl units to the triterpenoid intermediate squalene (SQ). The SQ in eukaryotes is biosynthesized by one squalene synthase (SQase), catalyzing a two-step reaction, including the condensation of two farnesyl diphosphate (FPP) to form presqualene diphosphate (PSPP) and the subsequent reductive rearrangement of PSPP to SQ [28]. By contrast, the SQ in prokaryotes is formed by a three-enzyme pathway using presqualene diphosphate synthase (HpnD), hydroxysqualene synthase (HpnC), and squalene/phytoene dehydrogenase (HpnE) (Figure 5, Table S6) [29]. The tandem cyclization of SQ is catalyzed by squalene–hopene cyclase (HpnF), thus providing the skeleton of triterpenoid. One notable feature of bacteriohopanetriols is the non-isopentenyl C5 side chain. The *S*-adenosyl-l-methionine (SAM)-dependent enzyme HpnH converts diploptene into adenosylhopane [23]. The adenosine part is cleaved by nucleoside phosphorylases (HpnG) into the phosphorylated ribose. Whether the reductive dephosphorylation is spontaneous or catalyzed by enzymes remains unknown. An aminotransferase, HpnO, is located in the cluster and is required in aminobacteriohopanetriol formation [30]. As previously reported, the post-tailoring steps on the terminal amino group can involve tryptophanyl, ornithinyl, and glycosyl [31]. The *N*-acetyl substituted product we obtained from ZS0520 was previously obtained by chemical synthesis [20]. Because of the examples of the substituents on the amino group, we speculate that the acetyl of compound **9** is catalyzed by an acetyltransferase. According to the bioinformatics analysis, the gene responsible for this acetyltransferase may not be encoded in the gene cluster, but it may be located somewhere else in the genome.

### 2.6. Proposed Biosynthetic Pathway of Minipyrone

**Figure 6:**
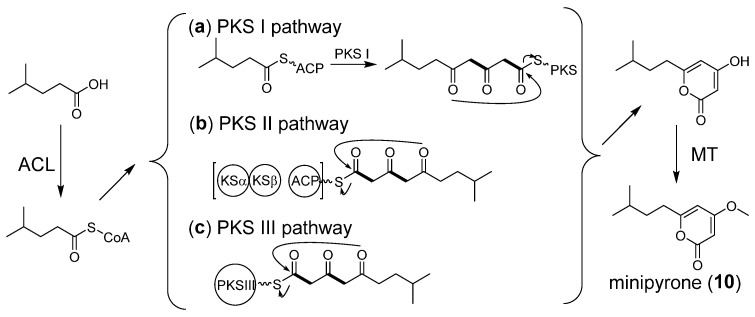
Proposed biosynthetic pathway of minipyrone.

Minipyrone is proposed to be produced via a PKS pathway. We propose that the biosynthetic pathway of minipyrone is similar to those of nocapyrones [32]. The genes encoding an acyl-CoA ligase (ACL), a PKS, and a methyltransferase (MT) should at least be harbored in the biosynthetic gene cluster. However, we searched all types of PKS clusters in the ZS0520 genome, and no candidate containing the required genes was found. We suppose the gene cluster is too short to be recognized. Through the PKS I pathway, a short-chain fatty acid could be activated by ACL to form the corresponding acyl-CoA and could then be transferred onto ACP (Figure 6a). A two-module PKS or an iterative PKS elongates the acyl-ACP using two malonyl-CoA units. The TE domain cyclizes and releases the polyketide chain to form α-pyrone. After being released from the PKS protein, the methyltransferase adds the methyl to OH-3, yielding minipyrone (**10**). Minipyrone can also be derived from the PKS II or PKS III pathway (Figure 6b,c). However, more in vivo evidence is required to identify the BGC of minipyrone.

## 3. Materials and Methods

### 3.1. General Experimental Procedures

IR spectra were collected using an IRAffinity-1 spectrometer (Shimadzu company, Kyoto, Japan) for KBr pellets. HRESIMS spectra were determined by a MaXis 4G UHR59 TOFMS spectrometer (Bruker Company, Karlsruhe, Germany). NMR spectra were recorded on a Bruker AVANCE III HD 700 spectrometer with tetramethylsilane (TMS) as the internal standard (Bruker Company, Karlsruhe, Germany). UV spectra were measured using a U-2600 spectrophotometer (Shimadzu Company, Kyoto, Japan).

Semi-preparative HPLC was carried out using the 1260 HPLC system equipped with a G1311C isocratic pump and an Agilent G1315D diode array detector (DAD) (Agilent Company, Santa Clara, CA, USA) using an ODS-A column (10 × 250 mm, 5 μm, YMC company, Kyoto, Japan). Column chromatographs were performed with silica gel (200–300 mesh; Yantai Jiangyou Silica Gel Development company, Yantai, CN), Sephadex LH-20 (GE Healthcare Company, Marlborough, MA, USA), and reversed-phase medium-pressure preparative liquid chromatography (RP-MPLC, Agela Company, Torrance, CA, USA).

### 3.2. Extraction and Purification

*Streptomyces* sp. SCSIO ZS0520 was cultured at 28 °C on agar plates with modified-MS medium. The mycelium was inoculated into 250 mL flasks containing 50 mL of modified-RA medium and incubated for 2 days at 28 °C on rotary shakers (200 rpm). After 2 days of growth, each seed culture was transferred equally to two 1 L flasks containing 200 mL of modified-RA medium. Flasks were fermented at 28 °C on rotary shakers (200 rpm) for 8 days. Approximately 40 L of fermentation broth was centrifuged (3900 rpm, 15 min) to separate the supernatant and mycelium. The supernatant was extracted three times with equal volumes of butanone, and the mycelium cake was extracted with acetone to obtain a crude extract. Finally, both extract residues were combined according to HPLC–DAD analysis.

The extract was concentrated at a reduced pressure to obtain 48 g of crude. The crude was subjected to silica gel CC. Using a gradient of CHCl_3_/MeOH (1:0–1:1, *v/v*), thirteen fractions (Fr. 1-1–13) were obtained according to the results of TLC. Fr. 6 was chromatographed using a Sephadex LH-20 CC eluted with CHCl_3_/MeOH (1:1, *v/v*) to yield four fractions (Fr. 6-1–6-4). Fr. 6-2 was subsequently chromatographed on silica gel CC and eluted with CHCl_3_/MeOH (100:1–1:1, *v/v*) to obtain Fr. 6-2-1–6-2-8. The mixture of compounds **2** and **3** (3 mg) was obtained by HPLC separation (MeCN/H_2_O) from Fr. 6-2-6. Compound **7** (39 mg) was purified using HPLC (MeCN/H_2_O) from Fr. 6-2-8. Fr. 7 and Fr. 8 were combined and subsequently chromatographed on a Sephadex LH-20 CC eluted with CHCl_3_/MeOH (1:1, *v/v*) to afford three fractions (Fr. 7-1–7-3). Compound **4** (3 mg) and the mixture of compounds **5** and **6** (3 mg) were obtained using HPLC (MeCN/H_2_O) from Fr. 7-2 and Fr. 7-3. Fr. 5 was chromatographed on a Sephadex LH-20 CC eluted with CHCl_3_/MeOH (1:1, *v/v*) to afford three fractions (Fr. 5-1–5-3). Compound **10** (3 mg) was yielded by HPLC (MeCN/H_2_O) from Fr. 5-3. Fr. 11 was subjected to Sephadex LH-20 to remove pigment before being subjected to silica gel CC to afford several parts (Fr. 11-1–11-7). Compounds **8** (31 mg) and **9** (2 mg) were purified by HPLC (MeCN/H_2_O) from Fr. 11-2 and Fr. 11-4, respectively.

Modified-MS medium: 2% mannitol, 2% soybean powder, 1.5% agar, 3% sea salt, and 0.2% CaCO_3_; pH 7.2–7.4.

Modified-RA medium: 2% soluble starch, 0.5% corn flour, 1% malt extract, 1% glucose, 1% maltose, 0.01% trace elements, 3% sea salt, and 0.2% CaCO_3_; pH 7.2–7.4.

### 3.3. Characterization of Compounds

*Seco*-salinomycins A and B (**2** and **3**): pale yellow amorphous powders; UV (MeOH) *λ*_max_ (log *ε*) 325 (2.28) nm; IR (film) *ν*_max_ 3389, 2932, 1699, 1456, 1018 cm^−1^; ^1^H and ^13^C NMR data, see Table S1; HRESIMS *m*/*z* 771.4644 [M + Na]^+^ (calcd. for C_42_H_68_O_11_Na, 771.4654).

*Seco*-salinomycin C (**4**): pale yellow amorphous powder; [*α*]$\overset{20}{\underset{D}{\;}}$ −71.0 (*c* 0.10, MeOH); UV (MeOH) *λ*_max_ (log *ε*) 315 (2.26) nm; ECD (*c* 0.025, MeOH) *λ*_max_ (Δ*ε*) 291 (−12.58), 328 (+2.36) nm; IR (film) *ν*_max_ 3385, 2933, 1645, 1456, 1020 cm^−1^; ^1^H and ^13^C NMR data, see Table S1; HRESIMS *m*/*z* 529.3135 [M + Na]^+^ (calcd. for C_29_H_46_O_7_Na, 529.3136).

*Seco*-salinomycins D and E (**5** and **6**): pale yellow amorphous powder; UV (MeOH) *λ*_max_ (log *ε*) 309 (2.15) nm; IR (film) *ν*_max_ 3460, 2967, 1699, 1456, 1049 cm^−1^; ^1^H and ^13^C NMR data, see Table S2; HRESIMS *m*/*z* 751.4970 [M − H]^−^ (calcd. for C_42_H_71_O_11_, 751.4996).

Minipyrone (**10**): yellow oil; UV (MeOH) *λ*_max_ (log *ε*) 282 (2.68) nm; IR (film) *ν*_max_ 3428, 2924, 1703, 1568, 1247 cm^−1^; ^1^H and ^13^C NMR data, see Table S2; HRESIMS *m*/*z* 197.1173 [M + H]^+^ (calcd. for C_11_H_17_O_3_, 197.1172).

### 3.4. Genome Sequencing and Analysis

The genome assembly and annotation methods were reported previously [2]. In brief, the genome DNA of ZS0520 was extracted using a Bacteria DNA Kit (OMEGA, Norcross, GA, USA). PacBio RS and Illumina sequencing technologies were used to scan the whole genome. The taxonomic classification of ZS0520 was built by querying all the genomes in the NCBI database using the MiGA website (http://microbial-genomes.org/, accessed on 9 April 2022). The comparative genomics graphs with ZS0520 and DSM 41398 were generated using MUMmer and LASTZ software. The collinear relationships between genomes were identified by MUMmer, while LASTZ was used to find the homologous regions in translocation, inversion, and translocation and inversion modes. The secondary metabolite BGCs were identified and analyzed using the antiSMASH 6.0.0alpha web tools (http://antismash.secondarymetabolites.org/, accessed on 19 October 2021). Gene annotation was achieved using BLAST (https://blast.ncbi.nlm.nih.gov/Blast.cgi, accessed on 19 October 2021).

## 4. Conclusions

In summary, the whole genome sequence of deep-sea hydrothermal vent-derived *Streptomyces* sp. SCSIO ZS0520 was acquired. Bioinformatic analysis revealed more than 34 biosynthetic clusters, implying its capacity to generate at least 34 classes of NPs. Among these BGCs, four distinct classes of metabolites (excluding the previously reported actinopyrone), including six new compounds, were isolated and characterized. Salinomycin, elaiophylin, and *N*-acetyl-aminobacteriohopanetriol are all potential antibiotics or ecological functional molecules. We proposed biosynthetic pathways for four types of secondary metabolites. This study might provide useful information not only for digging into the remaining biosynthetic problems of these compounds but also for mining the rest of the secondary metabolic gene clusters.

## Figures and Tables

**Table 1:** General genomic features of *Streptomyces* sp. SCSIO ZS0520.

Features	Value
Genome topology	linear
Assembly size (bp)	7,919,716
G + C content (%)	72.85
Coding DNA sequences	5956
tRNA genes	65
rRNA genes	18
Secondary metabolite gene clusters	34
GenBank accession	CP092644

## Data Availability

The authors declare that all relevant data supporting the findings of this study are available within the article and its Supplementary Material files or from the corresponding authors upon request.

## Supplementary Materials

The following supporting information can be downloaded at: https://www.mdpi.com/article/10.3390/md20060393/s1, Table S1, Table S2, Figure S1, and Figures S4–S35: ^1^H (700 MHz) and ^13^C NMR (175 MHz) data, HRESIMS spectra for compounds **2**–**6** and **10**; Table S3 and Figure S2: the BGCs and taxonomic classification of ZS0520 genome; Tables S4–S6: deduced function of *orfs* in three BGCs; Figure S3: the potential degradation mechanisms of salinomycin; Figure S36: the HPLC-DAD and HRESIMS spectra of crude extract.
