# Supplementary material for: Secondary Metabolites and Biosynthetic Gene Clusters Analysis of Deep-Sea Hydrothermal Vent-Derived Streptomyces sp. SCSIO ZS0520
Source: Mar Drugs. 2022 Jun 14;20(6):393. doi: 10.3390/md20060393 (PMC9228677; doi:10.3390/md20060393)
Supplement: Supplementary file 1 [file marinedrugs-20-00393-s001.zip › marinedrugs-1762508-supplementary.pdf]

Supporting Information

Secondary Metabolites and Biosynthetic Gene Clusters Analysis of Deep-sea Hydrothermal Vent-Derived *Streptomyces* sp. SCSIO ZS0520

Huaran Zhang [1,2,3], Yingying Chen [1], Yanqing Li [1,2,3], Yongxiang Song [1], Junying Ma [1,2,3] and Jianhua Ju [1,2,3*]

[1] Southern Marine Science and Engineering Guangdong Laboratory (Guangzhou), No.1119, Haibin Rd., Nansha District, Guangzhou 511458, China; aifyui@126.com (H.Z.); chenyingying7788@163.com (Y.C.); liyanqing20@mails.ucas.ac.cn (Y.L.); songx@scsio.ac.cn (Y.S.); majunying@scsio.ac.cn (J.M.)

[2] CAS Key Laboratory of Tropical Marine Bio-Resources and Ecology, Guangdong Key Laboratory of Marine Materia Medica, RNAM Center for Marine Microbiology, South China Sea Institute of Oceanology, Chinese Academy of Sciences, 164 West Xingang Road, Guangzhou 510301, China

[3] College of Oceanology, University of Chinese Academy of Sciences, Qingdao 266400, China

* Correspondence: jju@scsio.ac.cn (J.J.); Tel./Fax: +86-20-8902-3028

Table of Contents

Table S1. Summary of ^{1}H (700 MHz) and ^{13}C NMR (175 MHz) data for compounds **2**, **3**, and **4** (δ in ppm, J in Hz). 3

Table S2. Summary of ^{1}H (700 MHz) and ^{13}C NMR (175 MHz) data for compounds **5**, **6**, and **10** (δ in ppm, J in Hz). 4

Table S3. The antiSMASH-predicted BGCs for *Streptomyces* sp. SCSIO ZS0520 (the isolated compounds were marked by asterisks). 5

Table S4. Deduced functions of *orfs* in the *sln* BGC. 6

Table S5. Deduced functions of *orfs* in the *ela* BGC. 7

Table S6. Deduced functions of *orfs* in the *hpn* BGC. 8

Figure S1. The Selected HMBC and ^{1}H-^{1}H COSY of new compounds isolated from *Streptomyces* sp. SCSIO ZS0520. 9

Figure S2. The taxonomic classification of ZS0520 genome inferred by the Microbial Genomes Atlas (MiGA) found against all the genomes in the database. 10

Figure S3. The potential degradation mechanisms of salinomycin. 1

Figure S4. HRESIMS spectrum of *seco*-salinomycin A and B (**2** and **3**). 1

Figure S5. ^{1}H NMR (700 MHz, DMSO-d_6) spectrum of *seco*-salinomycin A and B (**2** and **3**). 1

Figure S6. ^{13}C NMR (175 MHz, DMSO-d_6) spectrum of *seco*-salinomycin A and B (**2** and **3**). 2

Figure S7. ^{13}C-DEPT (150 MHz, DMSO-d_6) spectrum of *seco*-salinomycin A and B (**2** and **3**). 2

Figure S8. ^{1}H–^{1}H COSY spectrum of *seco*-salinomycin A and B (**2** and **3**). 3

Figure S9. HSQC spectrum of *seco*-salinomycin A and B (**2** and **3**). 3

Figure S10. HMBC spectrum of *seco*-salinomycin A and B (**2** and **3**). 4

Figure S11. NOESY spectrum of *seco*-salinomycin A and B (**2** and **3**). 4

Figure S12. HRESIMS spectrum of *seco*-salinomycin C (**4**). 5

Figure S13. ^{1}H NMR (700 MHz, $CDCl_3$) spectrum of *seco*-salinomycin C (**4**). 5

Figure S14. ^{13}C NMR (175 MHz, $CDCl_3$) spectrum of *seco*-salinomycin C (**4**). 6

Figure S15. ^{13}C-DEPT (175 MHz, $CDCl_3$) spectrum of *seco*-salinomycin C (**4**). 6

Figure S16. ^{1}H–^{1}H COSY spectrum of *seco*-salinomycin C (**4**). 7

Figure S17. HSQC spectrum of *seco*-salinomycin C (**4**). 7

Figure S18. HMBC spectrum of *seco*-salinomycin C (**4**). 8

Figure S19. NOESY spectrum of *seco*-salinomycin C (**4**). 8

Figure S20. HRESIMS spectrum of *seco*-salinomycin D and E (**5** and **6**). 9

Figure S21. ^{1}H NMR (700 MHz, methanol-d_4) spectrum of *seco*-salinomycin D and E (**5** and **6**). 9

Figure S22. ^{13}C NMR (175 MHz, methanol-d_4) spectrum of *seco*-salinomycin D and E (**5** and **6**). 10

Figure S23. ^{13}C-DEPT (175 MHz, methanol-d_4) spectrum of *seco*-salinomycin D and E (**5** and **6**). 10

Figure S24. ^{1}H–^{1}H COSY spectrum of *seco*-salinomycin D and E (**5** and **6**). 11

Figure S25. HSQC spectrum of *seco*-salinomycin D and E (**5** and **6**). 11

Figure S26. HMBC spectrum of *seco*-salinomycin D and E (**5** and **6**). 12

Figure S27. NOESY spectrum of *seco*-salinomycin D and E (**5** and **6**). 12

Figure S28. HRESIMS spectrum of minipyrone (**10**). 13

Figure S29. ^{1}H NMR (700 MHz, methanol-d_4) spectrum of minipyrone (**10**). 13

Figure S30. ^{13}C NMR (175MHz, methanol-d_4) spectrum of minipyrone (**10**). 14

Figure S31. ^{13}C-DEPT NMR (175 MHz, methanol-d_4) spectrum of minipyrone (**10**). 14

Figure S32. ^{1}H–^{1}H COSY spectrum of minipyrone (**10**). 15

Figure S33. HSQC spectrum of minipyrone (**10**). 15

Figure S34. HMBC spectrum of minipyrone (**10**). 16

Figure S35. NOESY spectrum of minipyrone (**10**). 16

Figure S36. The HPLC-DAD and HRESIMS data of crude extract from *Streptomyces* sp. SCSIO ZS0520. 17

Table S1. Summary of ^{1}H (700 MHz) and ^{13}C NMR (175 MHz) data for compounds **2**, **3**, and **4** (δ in ppm, *J* in Hz).

No.	**2 and 3**[a]				**4**[b]	
	δ_H, mult. (*J* in Hz)	δ_C, type	δ_H, mult. (*J* in Hz)	δ_C, type	δ_H, mult. (*J* in Hz)	δ_C, type
1	-	178.9, C	-	179.0, C	-	180.0, C,
2	2.67, m	49.0, CH	2.67, m	48.9, CH	2.89, m	49.0, CH
3	3.70, m	75.4, CH	3.70, m	75.4, CH	4.02 dd (11.1, 6.0)	75.8, CH
4	*a* 1.68, m<br>*b* 1.33, m	20.2, CH_2	*a* 1.68, m<br>*b* 1.33, m	20.2, CH_2	*a* 1.45, m<br>*b* 1.90, m	19.8, CH_2
5	*a* 1.33, m<br>*b* 1.72, m	26.4, CH_2	*a* 1.33, m<br>*b* 1.72, m	26.4, CH_2	*a* 1.45, m<br>*b* 1.80, m	26.3, CH_2
6	1.72, m	27.1, CH	1.72, m	27.2, CH	1.80, m	28.2, CH
7	3.56, m	70.8, CH	3.56, m	70.7, CH	3.69 d (10.1, 1.7)	71.1, CH
8	1.33, m	36.7, CH	1.33, m	36.6, CH	1.49, m	36.5, CH
9	3.80, m	71.7, CH	3.80, m	71.7, CH	4.09, dd (10.2, 1.1)	69.8, CH
10	2.81, m	47.3, CH	2.81, m	47.1, CH	2.89, m	48.5, CH
11	-	216.0, C	-	215.6, C	-	215.4, C
12	2.92, m	54.2, CH	2.92, m	54.2, CH	2.89, m	54.9, CH
13	4.02, dd (8.9, 3.3)	75.9, CH	4.00, dd (8.9, 3.3)	75.9, CH	4.04, dd (7.1, 5.1)	76.4, CH
14	1.93, m	28.1, CH	1.93, m	28.1, CH	2.00, m	28.1, CH
15	*a* 2.40, m<br>*b* 1.72, m	33.3, CH_2	*a* 2.40, m<br>*b* 1.72, m	33.5, CH_2	*a* 2.33, dd (19.3, 6.2)<br>*b* 1.90, m	35.9, CH_2
16	-	119.5, C	-	119.1, C	-	120.5, C
17	-	142.2, C	-	142.2, C	-	143.3, C
18	7.32, d (15.2)	133.9, CH	7.33, d (15.2)	133.9, CH	7.23, d (14.9)	143.0, CH
19	6.78, d (15.2)	119.1, CH	6.78, d (15.2)	119.1, CH	6.43, dd (14.9, 8.4)	126.9, CH
20	-	196.8, C	-	196.5, C	9.56, d (8.4)	194.9, CH
21		105.9, C		105.6, C	1.83, s	17.9, CH_3
22	2.37, m	35.1, CH_2	2.37, m	34.4, CH_2	1.01, d (6.8)	17.8, CH_3
23	1.61, m	33.5, CH_2	1.61, m	34.9, CH_2	*a* 1.90, m<br>*b* 1.49, m	17.4, CH_2
24	-	87.0, C	-	86.7, C	0.85, t (7.4)	11.6, CH_3
25	3.25, m	72.9, CH	3.43, m	74.6, CH	0.87, d (7.0)	13.4, CH_3
26	1.63, m	20.2, CH_2	1.63, m	19.5, CH_2	0.74, d (6.9)	7.1, CH_3
27	*a* 1.47, m<br>*b* 1.41, m	29.9, CH_2	*a* 1.47, m<br>*b* 1.41, m	29.7, CH_2	0.95, d (5.0)	11.1, CH_3
28	-	70.0, C	-	70.1, C	*a* 1.49, m<br>*b* 1.32, m	22.5, CH_2
29	3.63, q (6.8)	76.3, CH	3.56, q (6.8)	76.1, CH	0.95, t (5.6)	12.2, CH_3
30	1.04, d (6.9)	15.4, CH_3	1.09, d (6.9)	15.1, CH_3		
31	1.15, q (7.5)	31.9, CH_2	1.18, q (7.5)	31.8, CH_2		
32	0.77, t (7.5)	6.9, CH_3	0.77, t (7.5)	6.9, CH_3		
33	1.19, s	23.8, CH_3	1.01, s	21.3, CH_3		
34	1.76, s	18.2, CH_3	1.76, s	18.3, CH_3		
35	0.91, d (7.1)	19.6, CH_3	0.90, d (7.1)	19.6, CH_3		
36	1.61, m	18.9, CH_2	1.61, m	18.9, CH_2		
37	0.75, t (7.1)	10.6, CH_3	0.75, t (7.1)	10.4, CH_3		
38	0.70, d (7.0)	13.8, CH_3	0.69, d (7.0)	13.8, CH_3		
39	0.61, d (6.6)	7.7, CH_3	0.60, d (6.6)	7.6, CH_3		
40	0.81, d (7.0)	11.6, CH_3	0.80, d (7.0)	11.6, CH_3		
41	*a* 1.33, m<br>*b* 1.59, m	22.9, CH_2	*a* 1.33, m<br>*b* 1.59, m	23.0, CH_2		
42	0.77, t (7.5)	12.7, CH_3	0.77, t (7.5)	12.7, CH_3		

[a]Measured in DMSO-d_6, [b]measured in $CDCl_3$;

Table S2. Summary of ^{1}H (700 MHz) and ^{13}C NMR (175 MHz) data for compounds **5**, **6**, and **10** (δ in ppm, J in Hz).

No.	**5**[a]		**6**[a]		**10**[a]	
	δ_H, mult. (J in Hz)	δ_C, type	δ_H, mult. (J in Hz)	δ_C, type	δ_H, mult. (J in Hz)	δ_C, type
1	-	179.2, C	-	179.2, C	-	167.8, C
2	2.91, m	51.0, CH	2.91, m	50.9, CH	6.02, d (2.2)	101.2, CH
3	3.94, m	77.2, C	3.94, m	77.2, C	-	174.0, C
4	*a* 1.78, m<br>*b* 1.45, m	20.9, CH_2	*a* 1.78, m<br>*b* 1.45, m	20.9, CH_2	5.52, d (2.2)	88, CH
5	*a* 1.91, m<br>*b* 1.47, m	27.3, CH_2	*a* 1.91, m<br>*b* 1.47, m	27.3, CH_2	-	167.7, C
6	1.83, m	29.6, CH	1.83, m	29.6, CH	2.52, t (7.9)	32.4, CH_2
7	3.78, m	72.7, CH	3.78, m	72.7, CH	1.55, m	36.9, CH_2
8	1.52, m	37.8, CH	1.52, m	37.8, CH	1.60, m	28.7, CH
9	4.09, dd (9.8, 6.6)	72.6, C	4.09, dd (9.8, 6.6)	72.5, C	0.95, d (6.5)	22.6, CH_3
10	2.95, m	48.9, CH	2.95, m	48.9, CH	0.95, d (6.5)	22.6, CH_3
11	-	217.4, C	-	217.5, C	3.84, s	56.9, CH_3
12	3.13, m	56.2, CH	3.13, m	56.2, CH		
13	5.33, dd (8.1, 3.7)	75.8, CH	5.33, dd (8.1, 3.7)	75.8, CH		
14	2.43, m	33.1, CH	2.43, m	33.1, CH		
15	*a* 2.73, m<br>*b* 2.26, m	46.2, CH_2	*a* 2.73, m<br>*b* 2.26, m	46.2, CH_2		
16	1.29, m	23.3, CH_2	1.29, m	23.3, CH_2		
17	-	169.9, C	-	172.2, C		
18	5.57, d (15.3)	112.4, CH	5.55, d (14.9)	112.1, CH		
19	7.71, dd (15.3, 11.6)	143.4, CH	7.45, dd (14.9, 12.2)	146.4, CH		
20	5.16, d (11.6)	96.4, CH	5.58, dt (12.2, 1.6)	97.9, CH		
21	-	169.0, C	-	162.9, C		
22	2.73, m	29.1, CH_2	2.73, m	29.1, CH_2		
23	*a* 1.78, m<br>*b* 1.69, m	32.1, CH_2	*a* 1.78, m<br>*b* 1.69, m	32.1, CH_2		
24	-	92.9, C	-	92.4, C		
25	3.60, dd (11.5, 2.5)	74.3, CH	3.64, dd (11.5, 2.5)	74.4, CH		
26	*a* 1.36, m<br>*b* 1.47, m	23.9, CH_2	*a* 1.36 m<br>*b* 1.47, m	23.9, CH_2		
27	*a* 1.69, m<br>*b* 1.65, m	30.6, CH_2	*a* 1.69, m<br>*b* 1.65, m	30.8, CH_2		
28	-	72.1, C	-	72.1, C		
29	3.81, q (7.3)	77.6, CH	3.81, q (7.3)	77.6, CH		
30	1.23, d (7.3)	15.1, CH_3	1.22, d (7.3)	15.0, CH_3		
31	1.31, q (7.5)	32.6, CH_2	1.30, q (7.5)	32.6, CH_2		
32	0.89, t (7.5)	6.7, CH_3	0.89, t (7.5)	6.7, CH_3		
33	1.33, s	22.2, CH_3	1.30, s	22.0, CH_3		
34	1.29, t	9.3, CH_3	1.29, t	9.3, CH_3		
35	0.88, d (7.0)	18.1, CH_3	0.88, d (7.0)	18.1, CH_3		
36	*a* 1.72, m<br>*b* 1.29, m	21.0, CH_2	*a* 1.72, m<br>*b* 1.29, m	21.0, CH_2		
37	0.83, t (7.3)	11.3, CH_3	0.83, t (7.3)	11.4, CH_3		
38	0.88, d (6.9)	14.1, CH_3	0.88, d (6.9)	14.1, CH_3		
39	0.79, d (7.0)	7.6, CH_3	0.78, d (7.0)	7.6, CH_3		
40	0.95, d (7.0)	11.6, CH_3	0.95, d (7.0)	11.6, CH_3		
41	*a* 1.36, m<br>*b* 1.47, m	22.7, CH_2	*a* 1.36, m<br>*b* 1.47, m	22.7, CH_2		
42	0.91, t (7.5)	12.6, CH_3	0.91, t (7.5)	12.6, CH_3		

[a]Measured in methanol-d_4

Table S3. The antiSMASH-predicted BGCs for *Streptomyces* sp. SCSIO ZS0520 (the isolated compounds were marked by asterisks).

BGC	Position		Type	Product
	From	To		
Cluster 1	17,846	63,286	NRPS	
Cluster 2	103,824	113,669	butyrolactone	
Cluster 3	113,958	230,585	Type I PKS	salinomycin*
Cluster 4	548,354	569,586	amglyccycl	
Cluster 5	645,592	808,502	Type I PKS, NRPS-like, PKS-like	
Cluster 6	853,021	884,112	terpene, RiPP-like	
Cluster 7	928,480	991,057	Lanthipeptide, NRPS	
Cluster 8	1,010,302	1,111,960	Type I PKS, NRPS-like	
Cluster 9	1,115,560	1,150,300	Thiopeptide	
Cluster 10	1,248,858	1,339,358	Type I PKS, terpene	actinopyrone*
Cluster 11	1,354,638	1,439,438	terpene, NRPS, lanthipeptide	
Cluster 12	1,556,179	1,567,289	siderophore	
Cluster 13	2,150,642	2,190,410	others	
Cluster 14	2,280,379	2,299,470	terpene	albaflavenone
Cluster 15	2,419,735	2,483,685	Type I PKS	
Cluster 16	2,513,091	2,535,635	lassopeptide	albusnodin
Cluster 17	3,106,467	3,114,851	butyrolactone	
Cluster 18	3,501,150	3,509,995	melanin	melanin
Cluster 19	3,624,813	3,671,426	Type I PKS	
Cluster 20	3,730,653	3,826,257	arylpolyene, NRPS-like, NRPS	
Cluster 21	4,311,222	4,353,560	NRPS-like, betalactone	
Cluster 22	4,401,222	4,422,674	lanthipeptide	
Cluster 23	4,798,159	4,833,944	non-alpha poly-amino acids	
Cluster 24	5,142,276	5,183,295	PKS-like, butyrolactone	
Cluster 25	5,346,951	5,369,557	lanthipeptide	SAL-2242
Cluster 26	5,409,264	5,477,405	NRPS-like, arylpolyene, other	
Cluster 27	5,589,266	5,694,891	hglE-KS, NRPS-like, Type II PKS, betalactone	
Cluster 28	6,136,116	6,179,921	Type I PKS	
Cluster 29	6,263,525	6,274,430	RiPP-like	
Cluster 30	6,274,677	6,319,284	ladderane, melanin	melanin
Cluster 31	6,655,575	6,665,973	ectoine	ectoine
Cluster 32	7,514,085	7,540,764	terpene	*N*-acetyl-aminobacteriohopanetriol*
Cluster 33	7,695,695	7,709,539	siderophore	
Cluster 34	7,743,446	7,904,045	Type I PKS, phenazine, NRPS	elaiophylin*

Table S4. Deduced functions of *orfs* in the *sln* BGC.

orfs	Size	Proposed function	ID/SI	Protein homologue and origin
1	572	3-hydroxybutyryl-CoA dehydrogenase	100/100	(AEZ53943.1): *Streptomyces albus* XM211
2	286	3-oxoacyl-(acyl-carrier-protein) synthase III	99/100	(AEZ53944.1): *Streptomyces albus* XM211
A1	4897	polyketide synthase	99/99	(AEZ53945.1): *Streptomyces albus* XM211
A2	3917	polyketide synthase	99/99	(AEZ53946.1): *Streptomyces albus* XM211
A3	2698	polyketide synthase	99/99	(AEZ53947.1): *Streptomyces albus* XM211
A4	1645	polyketide synthase	99/99	(AEZ53948.1): *Streptomyces albus* XM211
A5	3750	polyketide synthase	99/99	(AEZ53949.1): *Streptomyces albus* XM211
A6	1435	polyketide synthase	99/99	(AEZ53950.1): *Streptomyces albus* XM211
A7	1644	polyketide synthase	99/99	(AEZ53951.1): *Streptomyces albus* XM211
A8	3724	polyketide synthase	99/99	(AEZ53952.1): *Streptomyces albus* XM211
A9	2312	polyketide synthase	99/100	(AEZ53953.1): *Streptomyces albus* XM211
B1	128	epoxide hydrolase	100/100	(AEZ53954.1): *Streptomyces albus* XM211
D1	265	thioesterase	99/99	(AEZ53955.1): *Streptomyces albus* XM211
M	271	*O*-methyltransferase-like protein	99/99	(AEZ53956.1): *Streptomyces albus* XM211
E	93	ferredoxin protein	98/98	(AEZ53957.1): *Streptomyces albus* XM211
F	393	cytochrome P450	100/100	(AEZ53958.1): *Streptomyces albus* XM211
TI	325	ABC transporter ATP-binding protein	97/97	(AEZ53959.1): *Streptomyces albus* XM211
TII	546	antibiotic ABC transporter efflux pump	99/99	(AEZ53960.1): *Streptomyces albus* XM211
BII	149	epoxide hydrolase	100/100	(AEZ53961.1): *Streptomyces albus* XM211
BIII	153	epoxide hydrolase	99/99	(AEZ53962.1): *Streptomyces albus* XM211
C	484	epoxidase	99/99	(AEZ53963.1): *Streptomyces albus* XM211
R	907	transcriptional regulator	99/99	(AEZ53964.1): *Streptomyces albus* XM211
DII	239	thioesterase	99/100	(AEZ53965.1): *Streptomyces albus* XM211
3	597	peptide synthetase	99/100	(AEZ53966.1): *Streptomyces albus* XM211
4	74	hypothetical protein	100/100	(AJE80634.1): *Streptomyces albus*
5	73	hypothetical protein	100/100	(AJE80633.1): *Streptomyces albus*
6	78	hypothetical protein	100/100	(AEZ53967.1): *Streptomyces albus* XM211
7	855	SARP family transcriptional regulator	99/98	(AEZ53968.1): *Streptomyces albus* XM211
8	597	AMP-binding domain-containing protein	99/99	(AEZ53969.1): *Streptomyces albus* XM211
9	572	acyl-CoA dehydrogenase	97/97	(AEZ53970.1): *Streptomyces albus* XM211
10	96	peptide carrier protein	100/100	(AEZ53971.1): *Streptomyces albus* XM211

Table S5. Deduced functions of *orfs* in the *ela* BGC.

orfs	Size	Proposed function	ID/SI	Protein homologue and origin
elaA	799	LuxR family transcriptional regulator	41/54	(AKZ60150.1): *Streptomyces ambofaciens* ATCC 23877
elaB	486	dTDP-4-keto-6-deoxy-hexose 2,3-dehydratase	45/61	(AJO72724.1): *Nocardia brasiliensis* IFM-0406
elaC	328	dTDP-4-keto-6-deoxy-hexose 2,3-reductase	69/79	(AJO72719.1): *Nocardia brasiliensis* IFM-0406
elaD	341	dTDP-4-keto-6-deoxy-hexose 4-ketoreductase	46/60	(AJO72721.1): *Nocardia brasiliensis* IFM-0406
elaE	216	LuxR family two component transcriptional regulator	99/99	(AJE87692.1): *Streptomyces albus* DSM 41398
elaF	461	sensor histidine kinase	97/96	(AJE87694.1): *Streptomyces albus* DSM 41398
elaG	245	ABC transporter	100/100	(AJE87693.1): *Streptomyces albus* DSM 41398
elaH	302	ABC transporter	100/100	(AJE87695.1): *Streptomyces albus* DSM 41398
elaI	209	dTDP-4-keto-6-deoxy-hexose 3,5-epimerase	52/65	(AJO72701.1): *Nocardia brasiliensis* IFM-0406
elaJ	417	glycosyltransferase	38/57	(AJO72738.1): *Nocardia brasiliensis* IFM-0406
elaK	261	thioesterase	99/100	(AJE87697.1): *Streptomyces albus* DSM 41398
elaL	2086	polyketide synthase	99/99	(AJE87699.1): *Streptomyces albus* DSM 41398
elaM	3411	polyketide synthase	99/99	(AJE87700.1): *Streptomyces albus* DSM 41398
elaN	1631	polyketide synthase	99/99	(AJE87701.1): *Streptomyces albus* DSM 41398
elaO	1622	polyketide synthase	99/99	(AJE87702.1): *Streptomyces albus* DSM 41398
elaP	4367	polyketide synthase	99/99	(AJE87703.1): *Streptomyces albus* DSM 41398
elaQ	320	dTDP-glucose 4,6-dehydratase	100/100	(AJE87704.1): *Streptomyces albus* DSM 41398
elaR	274	glucose-1-phosphate thymidylyltransferase	54/71	(AJO72746.1): *Nocardia brasiliensis* IFM-0406
elaS	1127	LuxR family transcriptional regulator	93/94	(AJE87706.1): *Streptomyces albus* DSM 41398

Table S6. Deduced functions of *orfs* in the *hpn* BGC.

orfs	Size	Proposed function	ID/SI	Protein homologue and origin
hpnC	301	hydroxysqualene synthase	31/45	(CAA04732.1): *Zymomonas mobilis*
hpnD	312	presqualene diphosphate synthase	39/52	(CAA04733.1): *Zymomonas mobilis*
hpnE	478	squalene/phytoene dehydrogenase	28/41	(CAA04734.1): *Zymomonas mobilis*
hpnB	342	polyprenyl synthase	56/67	(ADO85577.1): *Streptomyces arenae*
hpnF	657	squalene-hopene cyclase	41/58	(CAA04735.1): *Zymomonas mobilis*
hpnG	217	nucleoside phosphorylases	73/81	(CAB39698.1): *Streptomyces coelicolor* A3(2)
hpnH	337	radical SAM enzyme	92/97	(CAB39699.1): *Streptomyces coelicolor* A3(2)
hpnO	461	aminotransferase	87/92	(CAB39702.1): *Streptomyces coelicolor* A3(2)

Figure S1. The Selected HMBC and ^{1}H-^{1}H COSY of new compounds isolated from *Streptomyces* sp. SCSIO ZS0520.

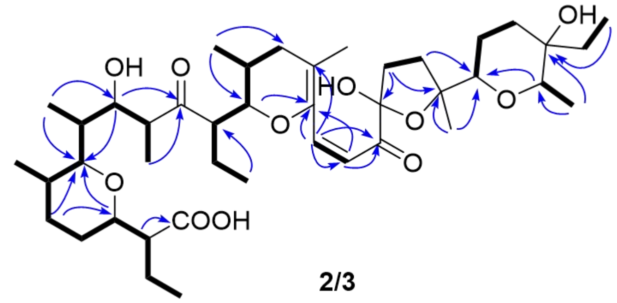

2/3

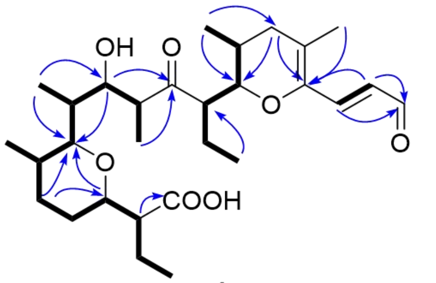

4

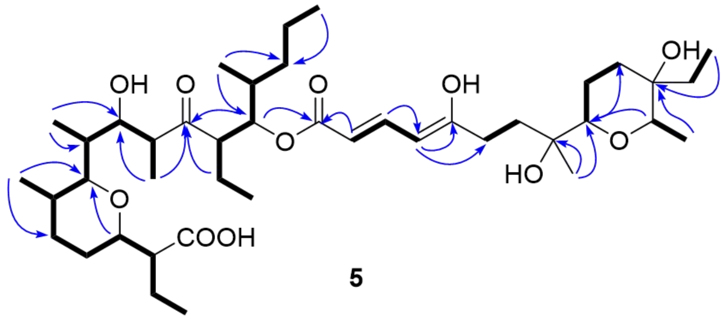

5

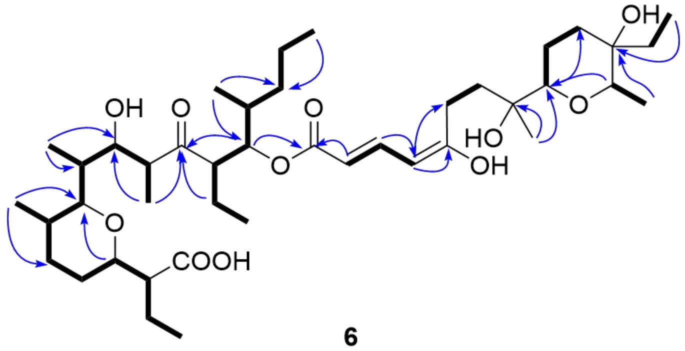

6

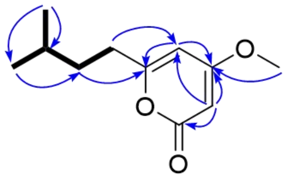

10

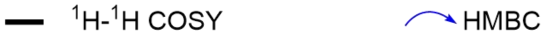

Figure S2. The taxonomic classification of ZS0520 genome inferred by the Microbial Genomes Atlas (MiGA) found against all the genomes in the database.

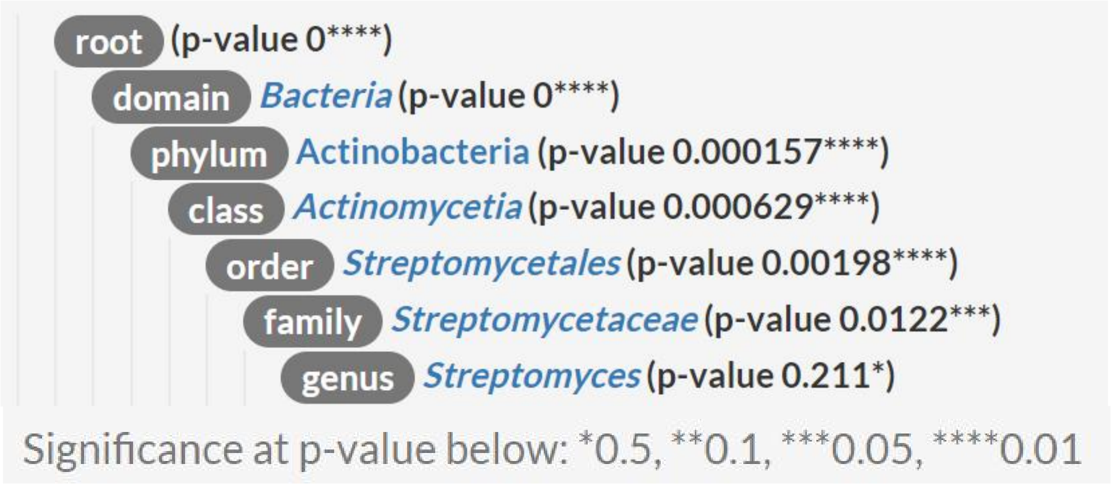

Figure S3. The potential degradation mechanisms of salinomycin.

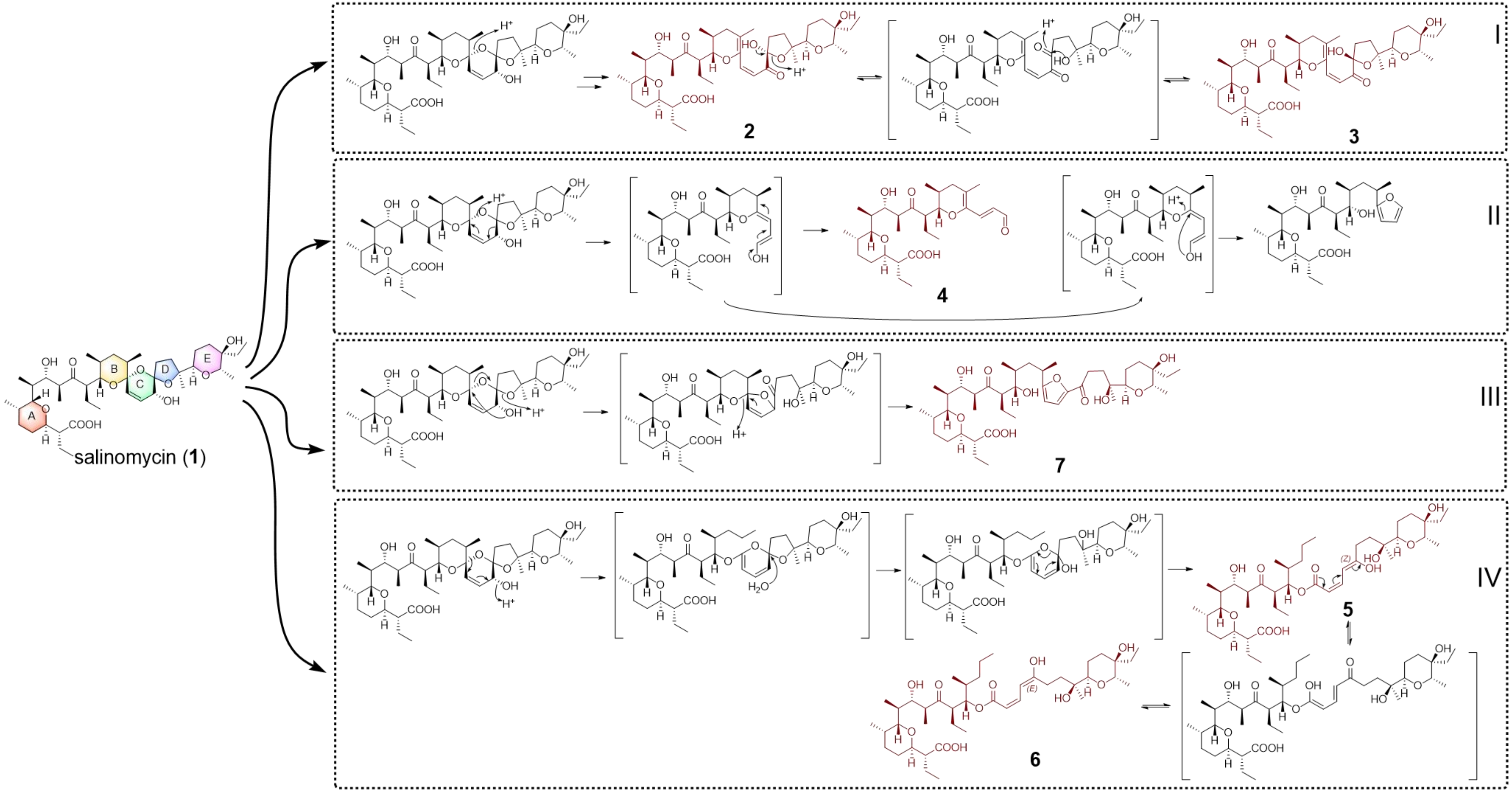

Figure S4. HRESIMS spectrum of *seco*-salinomycin A and B (**2** and **3**).

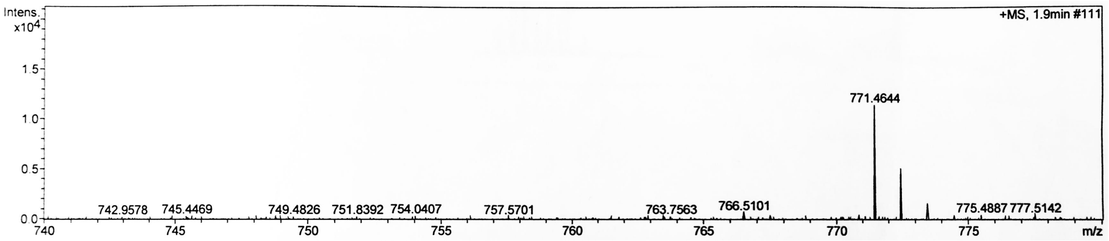

Figure S5. ^{1}H NMR (700 MHz, DMSO-d_6) spectrum of *seco*-salinomycin A and B (**2** and **3**).

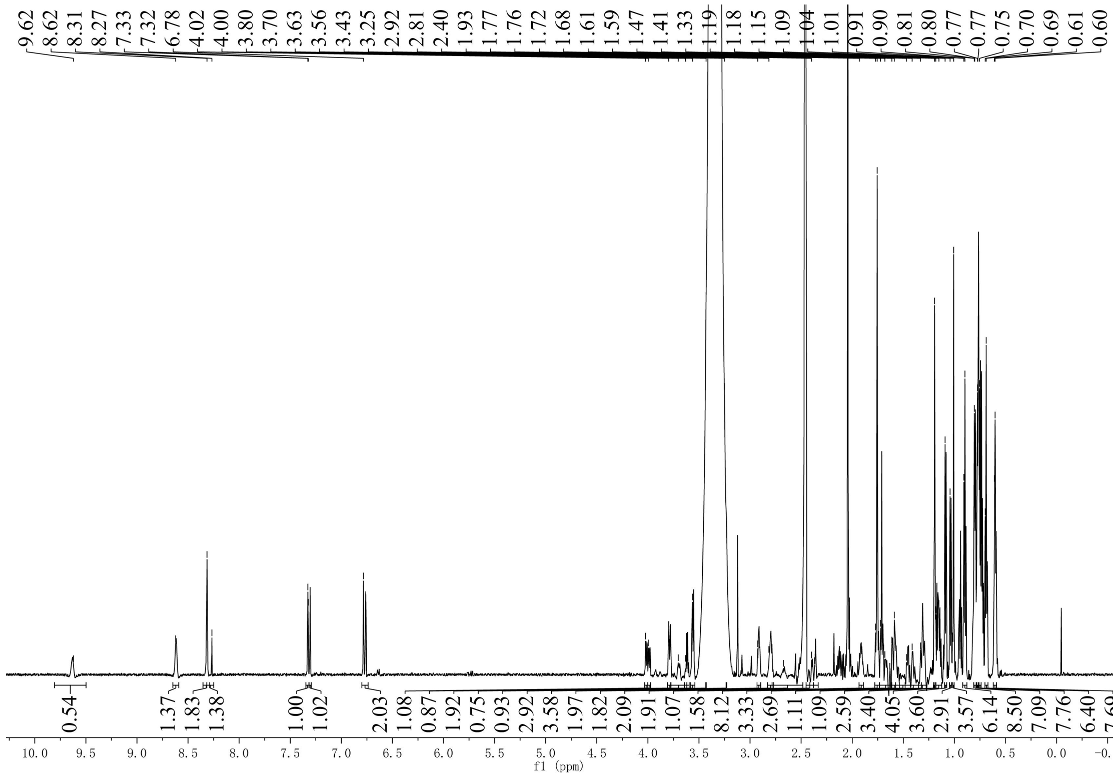

Figure S6. ^{13}C NMR (175 MHz, DMSO-d_6) spectrum of *seco*-salinomycin A and B (**2** and **3**).

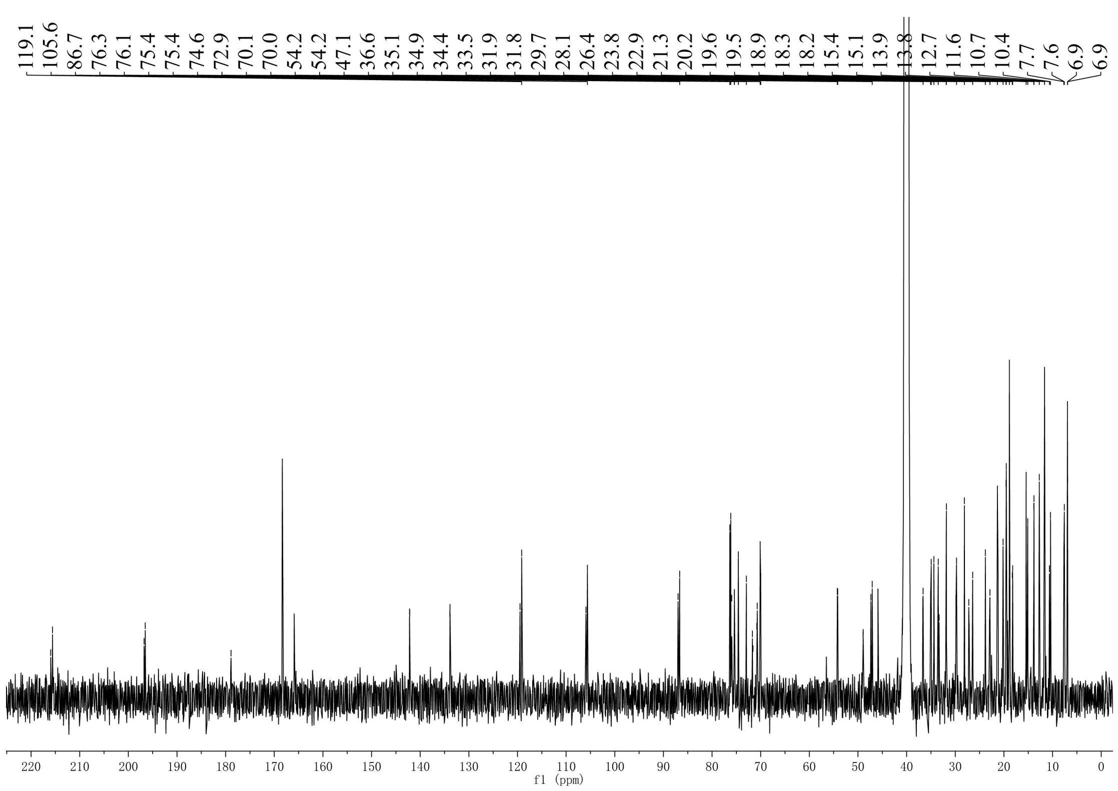

Figure S7. ^{13}C-DEPT (150 MHz, DMSO-d_6) spectrum of *seco*-salinomycin A and B (**2** and **3**).

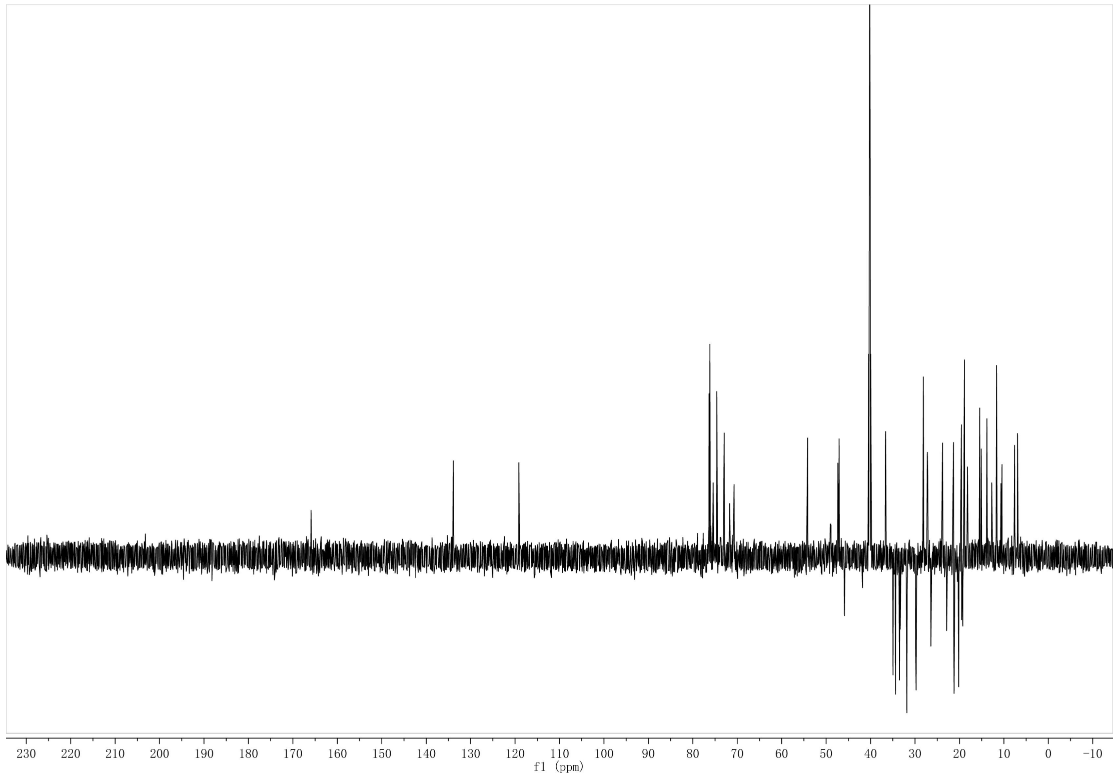

Figure S8. ^{1}H–^{1}H COSY spectrum of *seco*-salinomycin A and B (**2** and **3**).

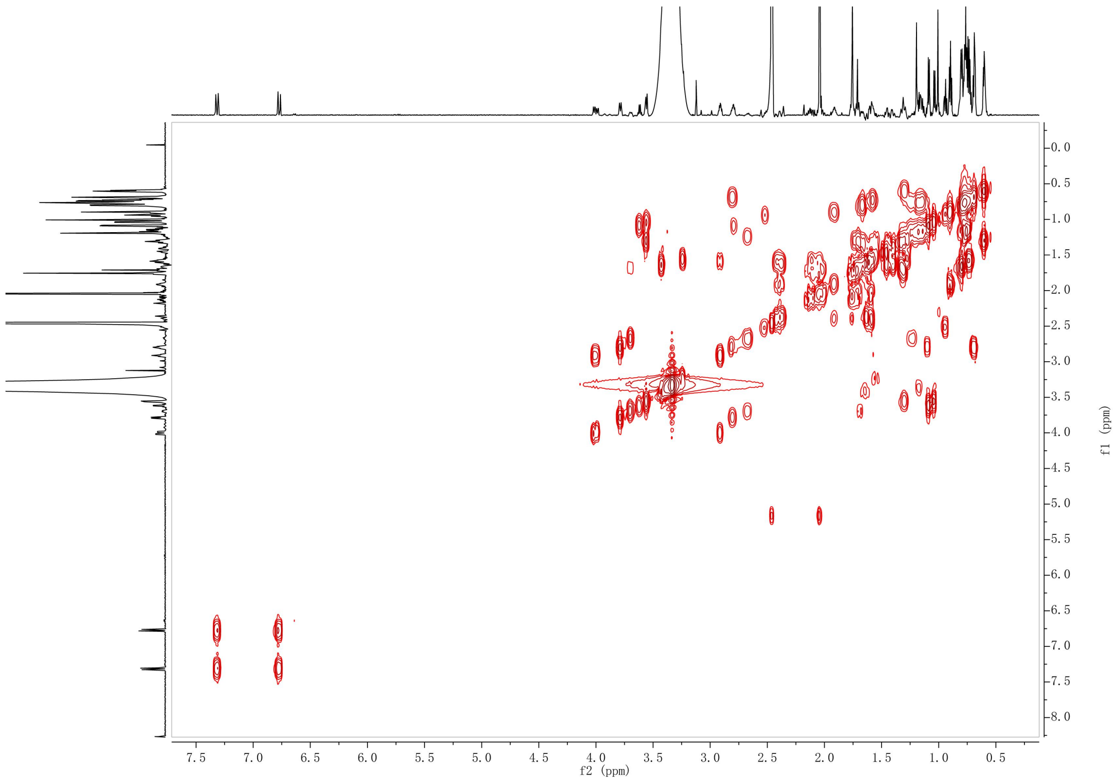

Figure S9. HSQC spectrum of *seco*-salinomycin A and B (**2** and **3**).

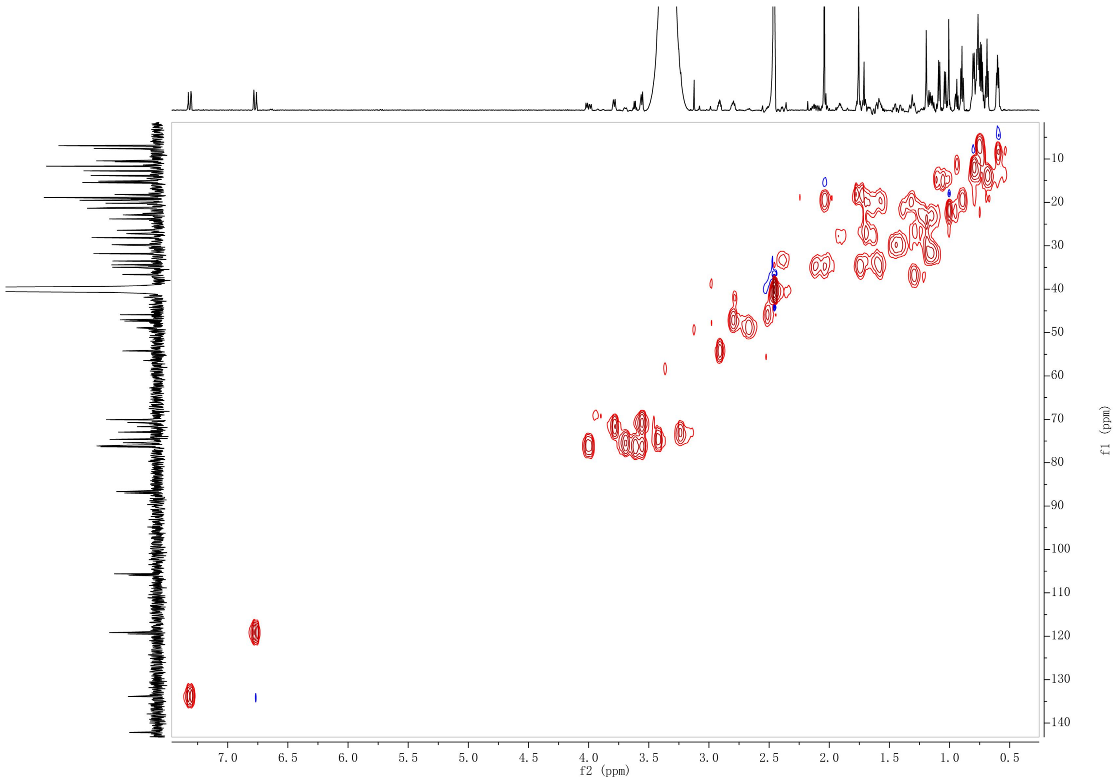

Figure S10. HMBC spectrum of *seco*-salinomycin A and B (**2** and **3**).

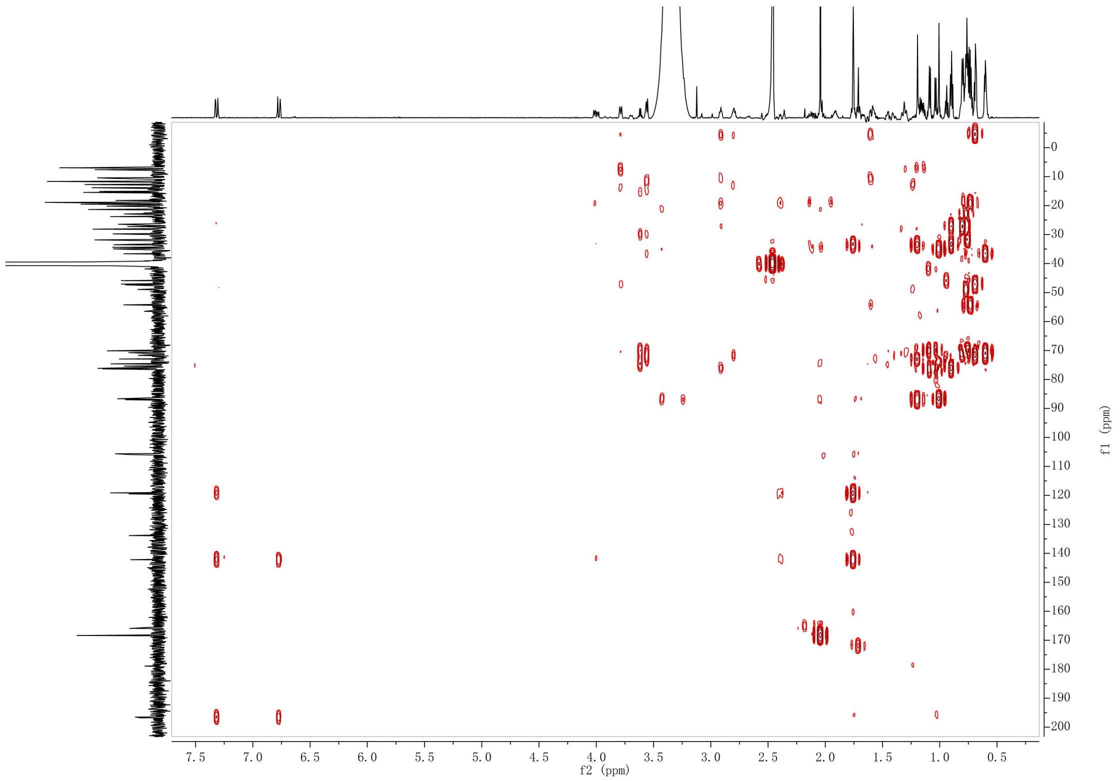

Figure S11. NOESY spectrum of *seco*-salinomycin A and B (**2** and **3**).

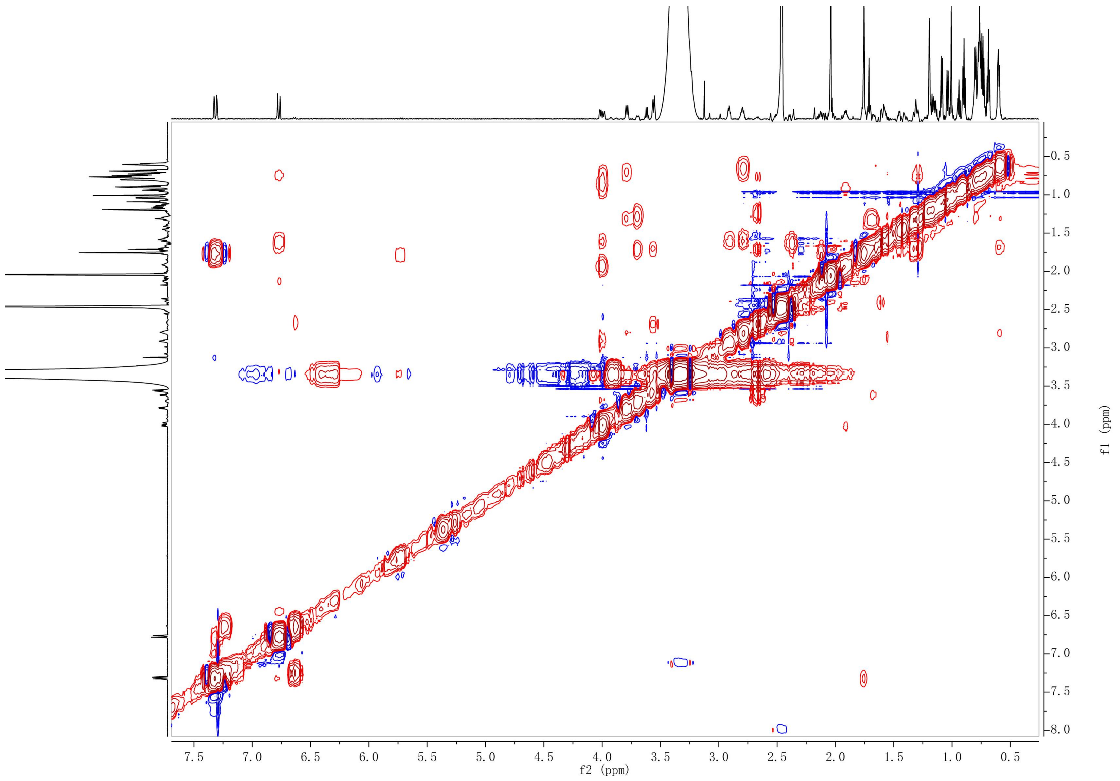

Figure S12. HRESIMS spectrum of *seco*-salinomycin C (**4**).

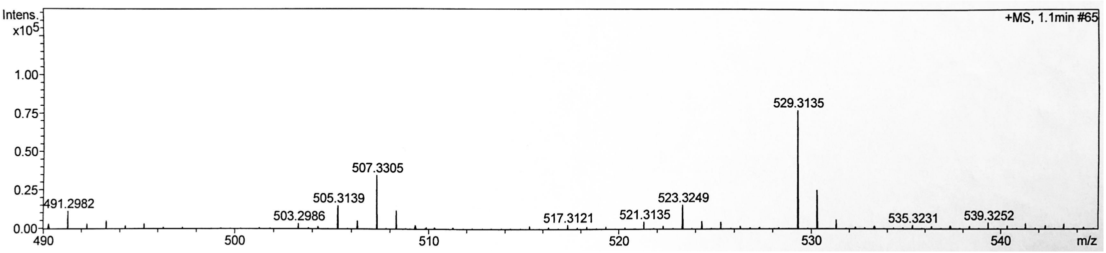

Figure S13. ^{1}H NMR (700 MHz, $CDCl_3$) spectrum of *seco*-salinomycin C (**4**).

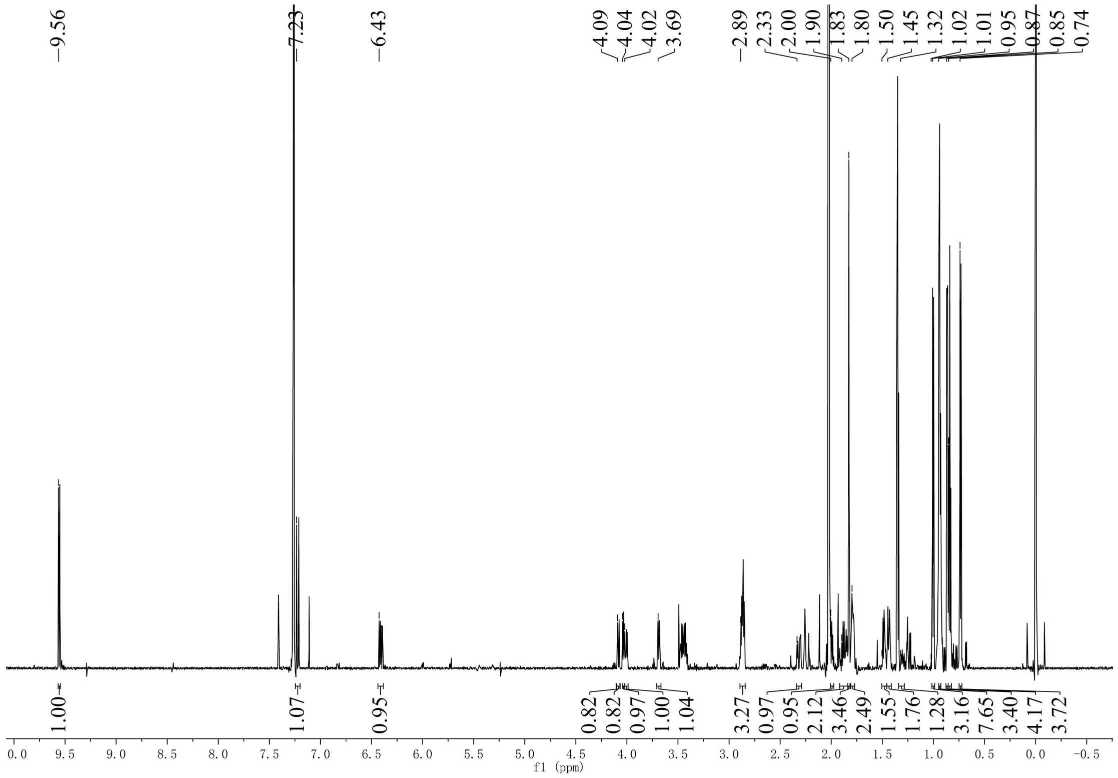

Figure S14. ^{13}C NMR (175 MHz, $CDCl_3$) spectrum of *seco*-salinomycin C (**4**).

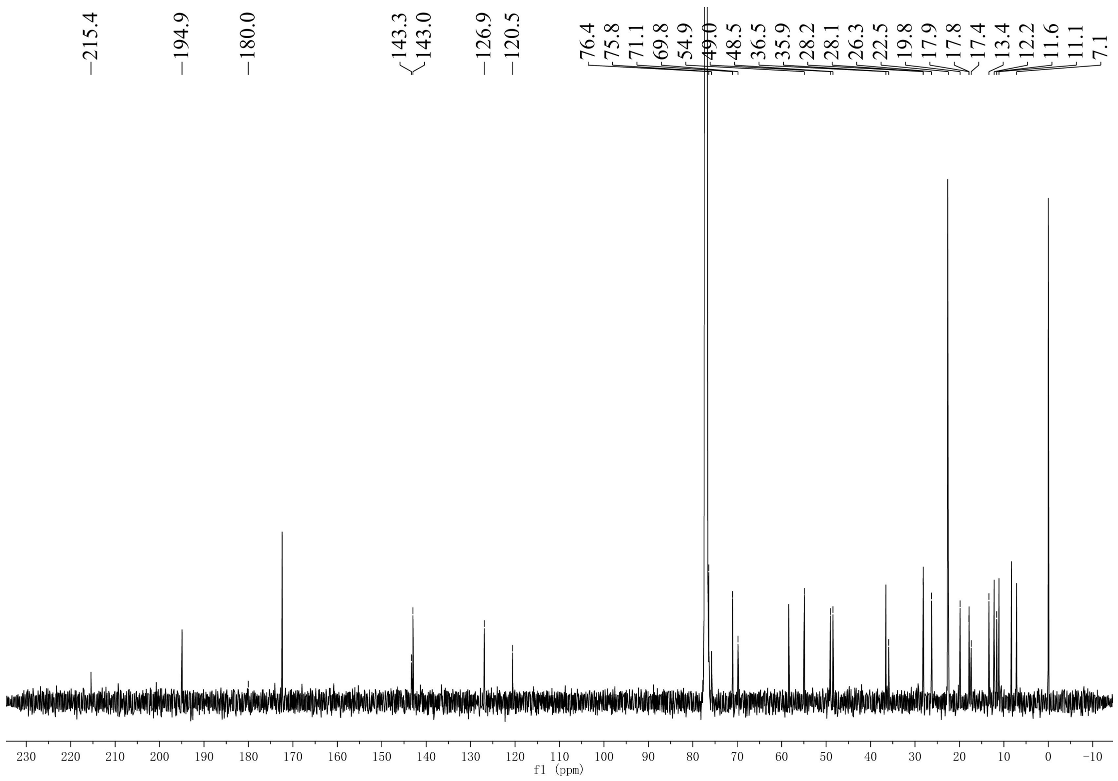

Figure S15. ^{13}C-DEPT (175 MHz, $CDCl_3$) spectrum of *seco*-salinomycin C (**4**).

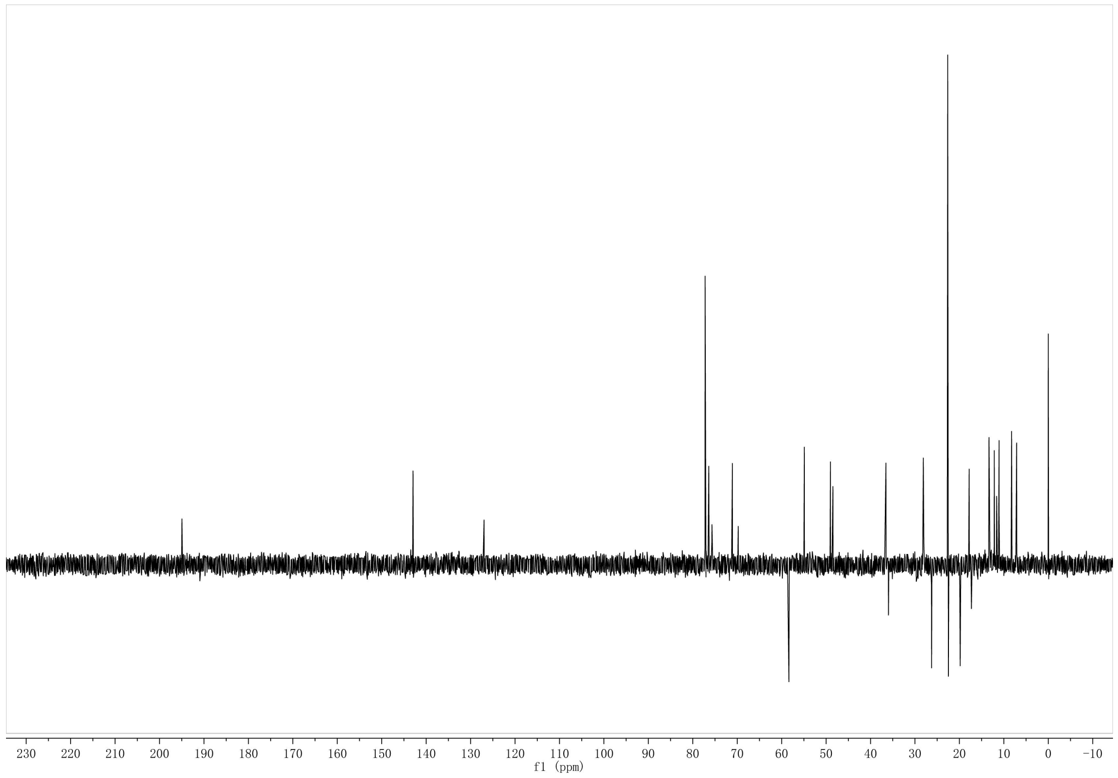

Figure S16. ¹H–¹H COSY spectrum of *seco*-salinomycin C (**4**).

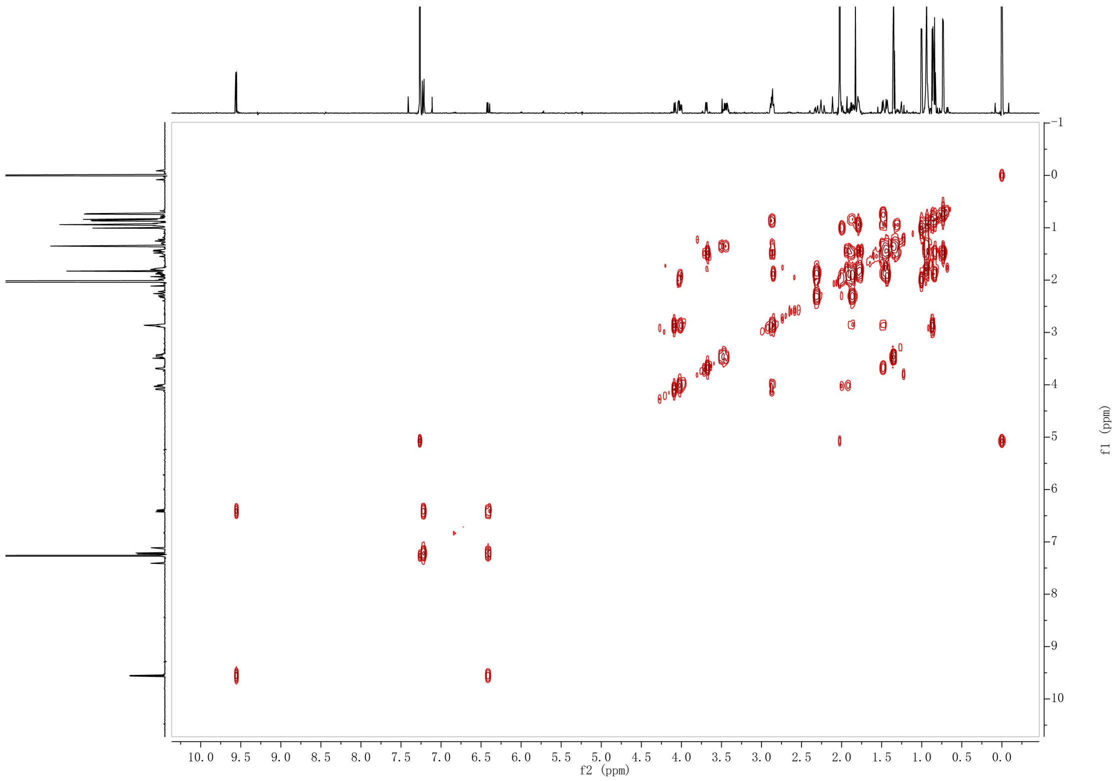

Figure S17. HSQC spectrum of *seco*-salinomycin C (**4**).

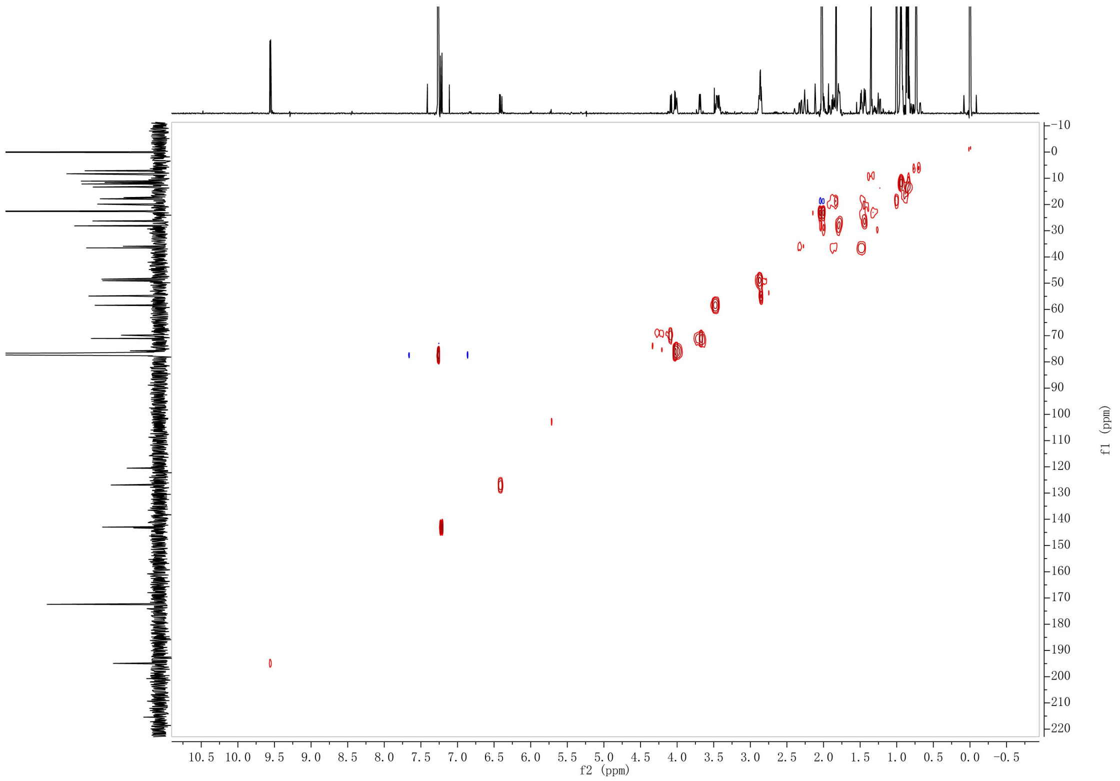

Figure S18. HMBC spectrum of *seco*-salinomycin C (**4**).

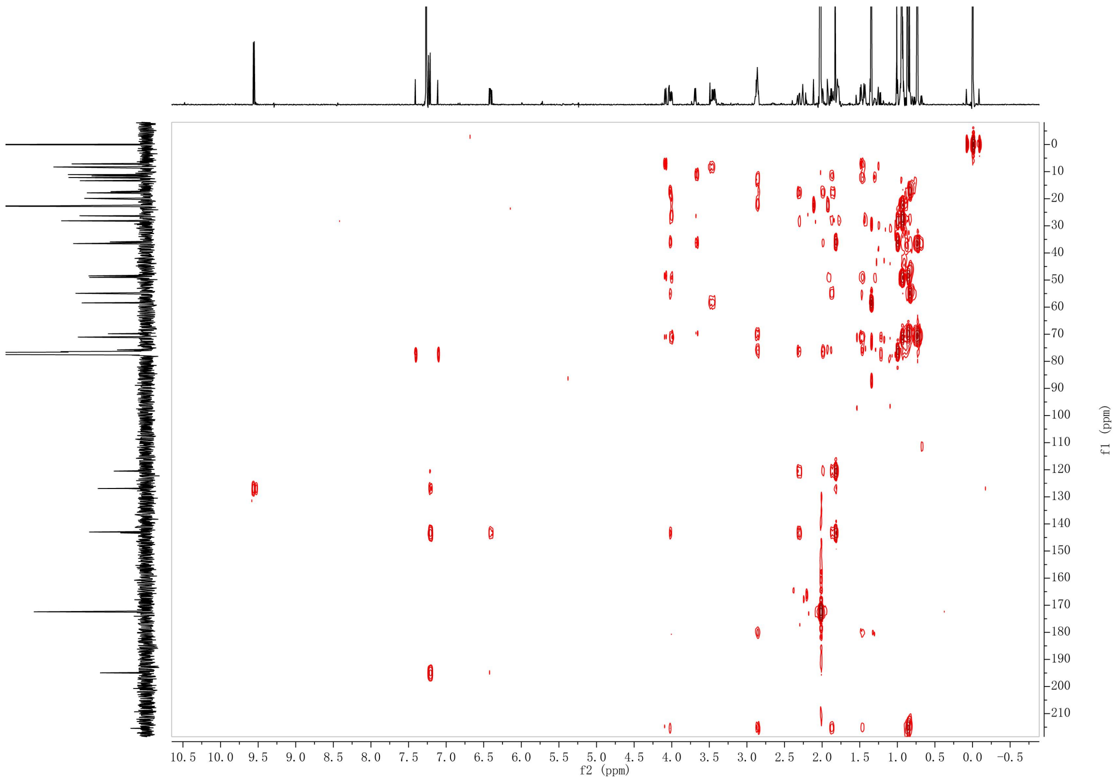

Figure S19. NOESY spectrum of *seco*-salinomycin C (**4**).

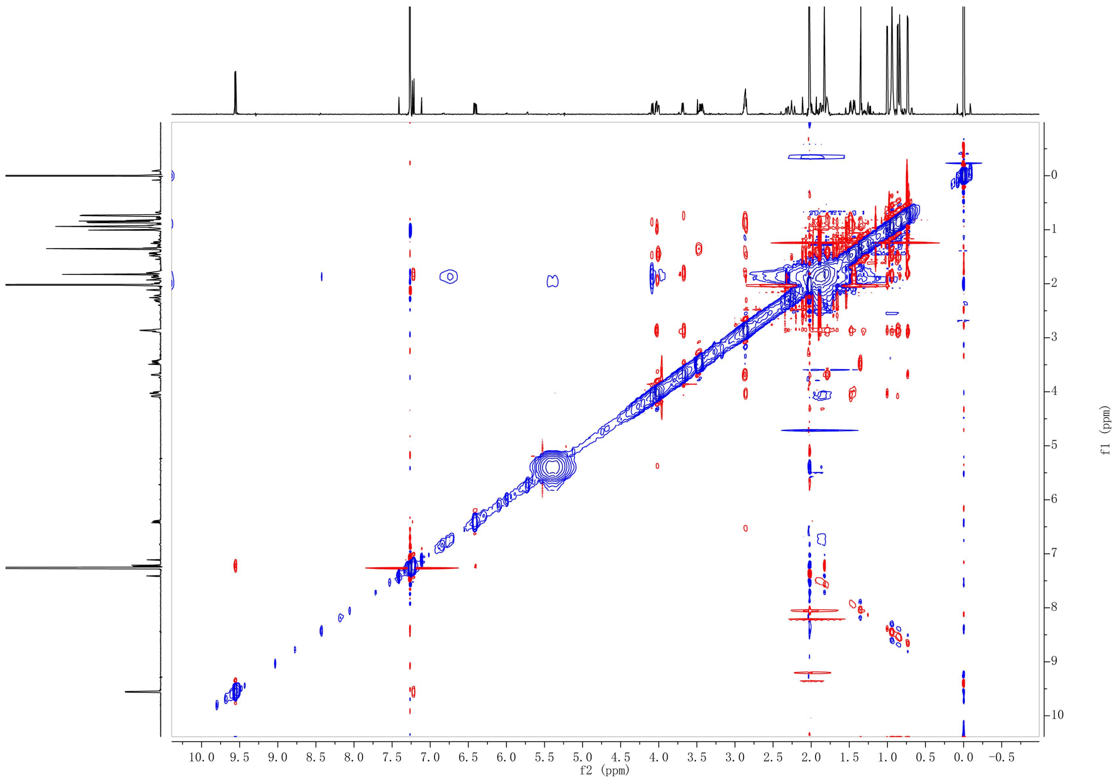

Figure S20. HRESIMS spectrum of *seco*-salinomycin D and E (**5** and **6**).

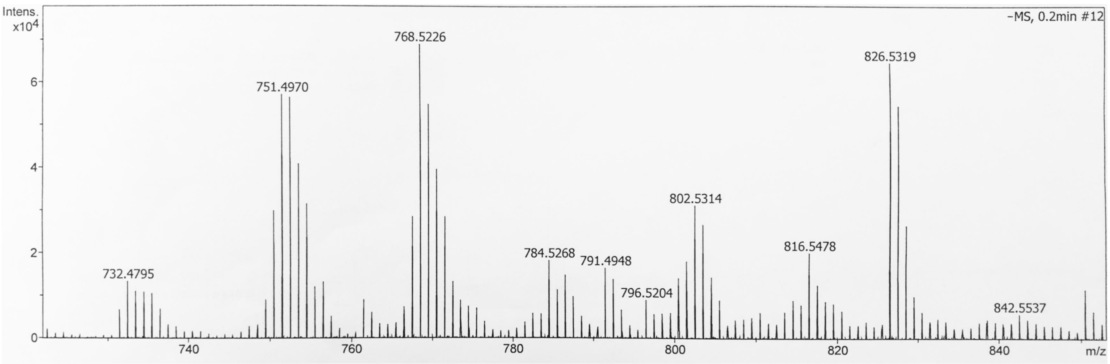

Figure S21. ^{1}H NMR (700 MHz, methanol-d_4) spectrum of *seco*-salinomycin D and E (**5** and **6**).

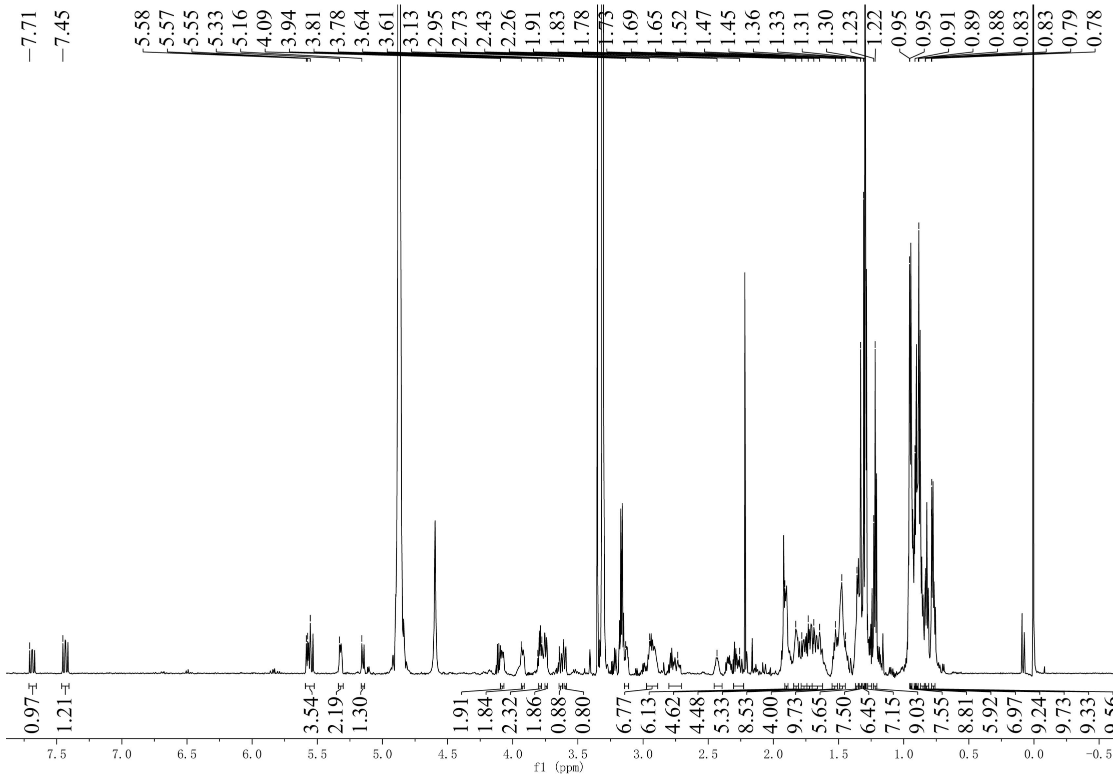

Figure S22. ^{13}C NMR (175 MHz, methanol-d_4) spectrum of *seco*-salinomycin D and E (**5** and **6**).

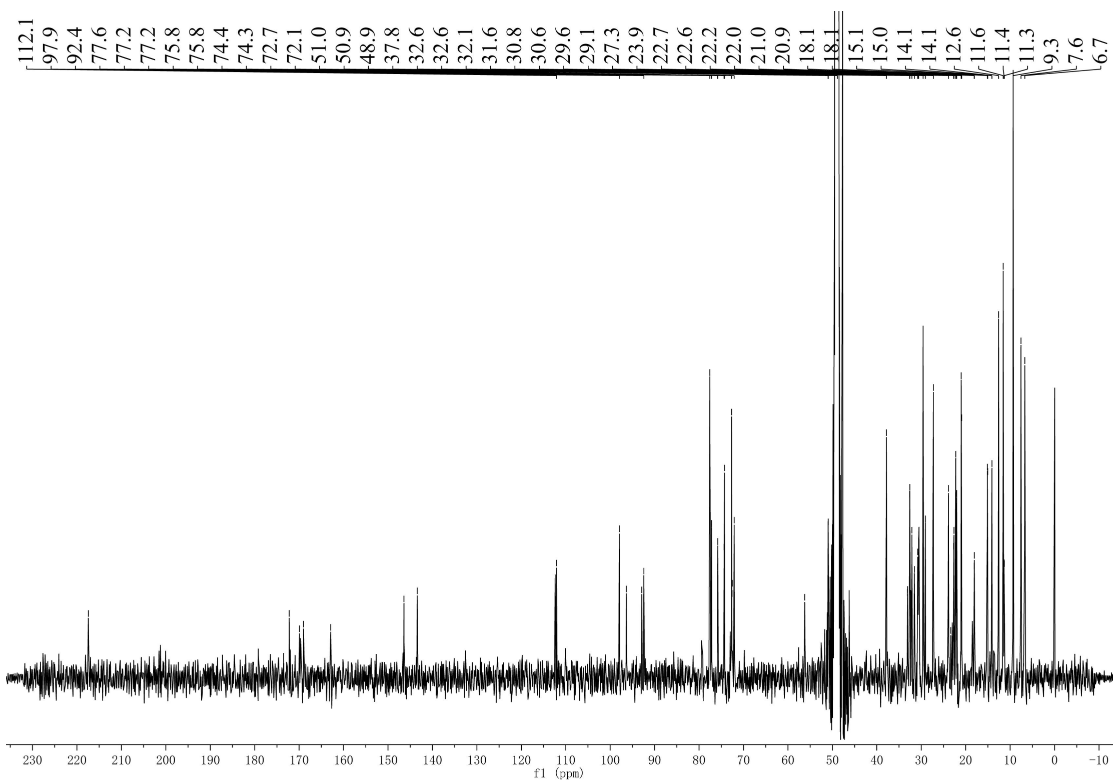

Figure S23. ^{13}C-DEPT (175 MHz, methanol-d_4) spectrum of *seco*-salinomycin D and E (**5** and **6**).

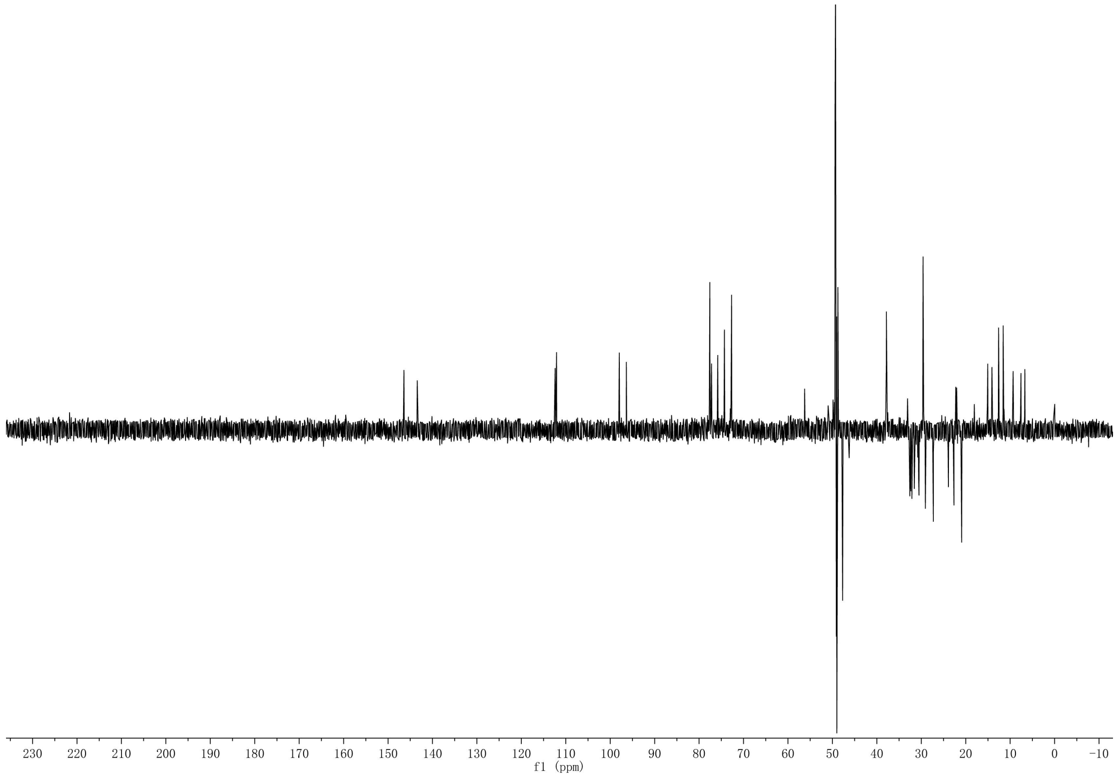

Figure S24. ^{1}H–^{1}H COSY spectrum of *seco*-salinomycin D and E (**5** and **6**).

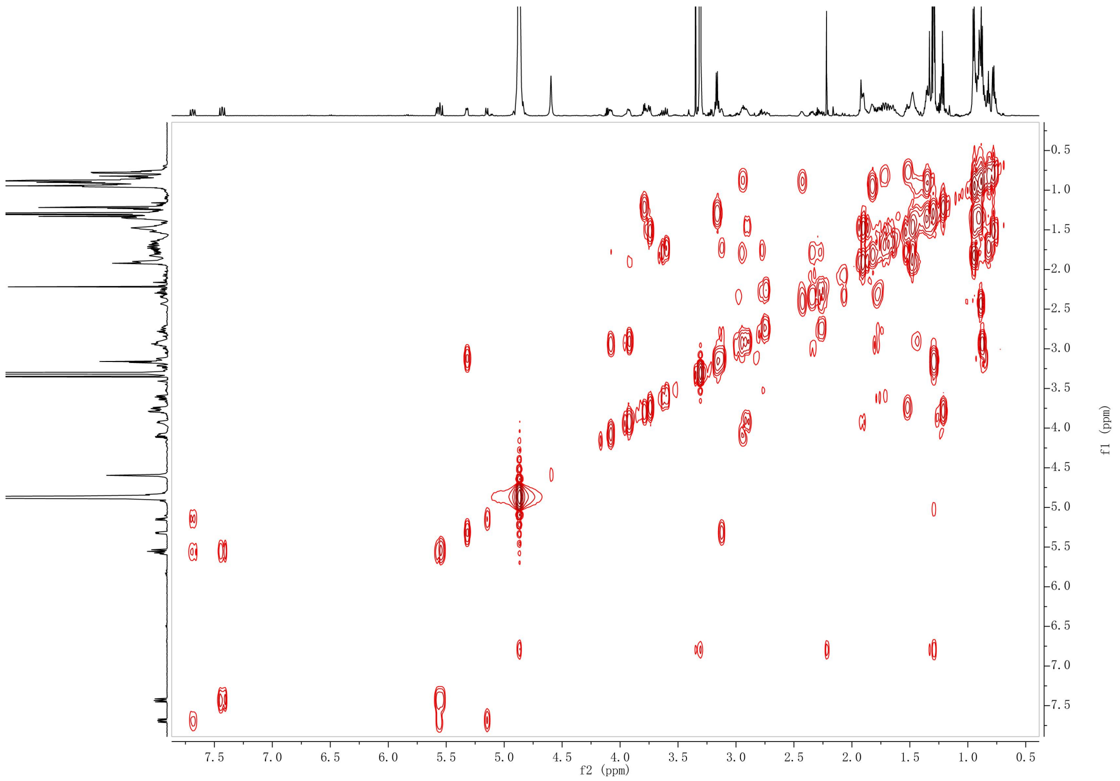

Figure S25. HSQC spectrum of *seco*-salinomycin D and E (**5** and **6**).

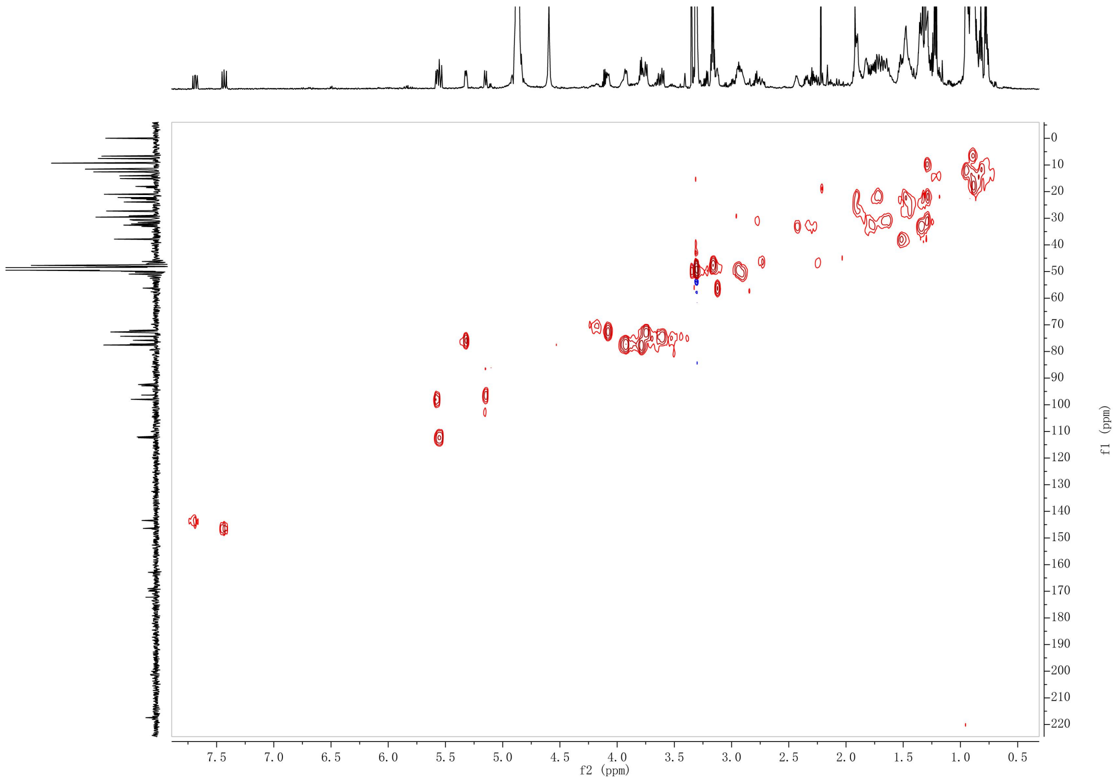

Figure S26. HMBC spectrum of *seco*-salinomycin D and E (**5** and **6**).

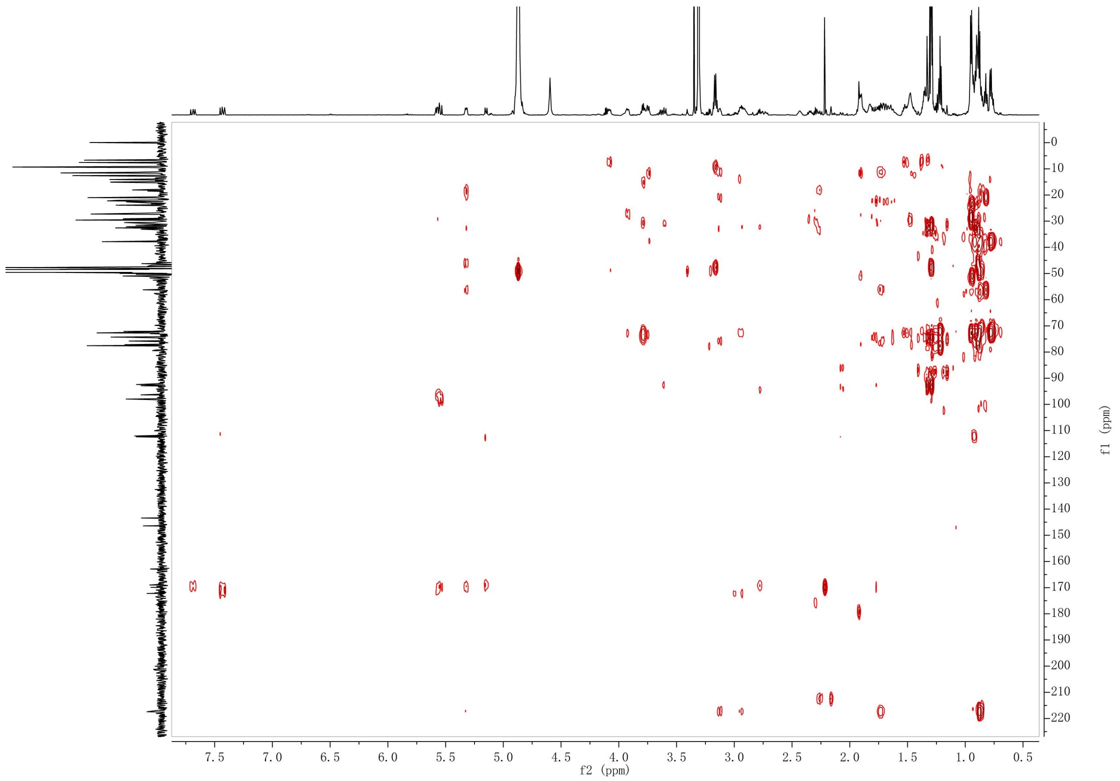

Figure S27. NOESY spectrum of *seco*-salinomycin D and E (**5** and **6**).

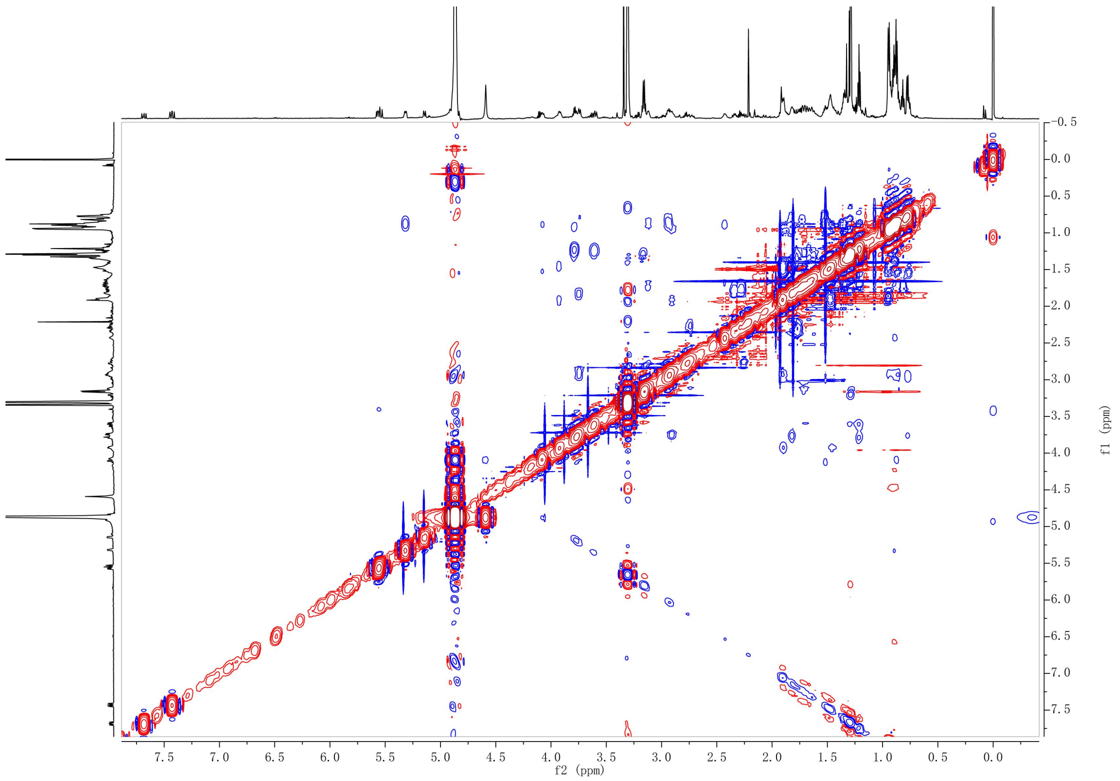

Figure S28. HRESIMS spectrum of minipyrone (**10**).

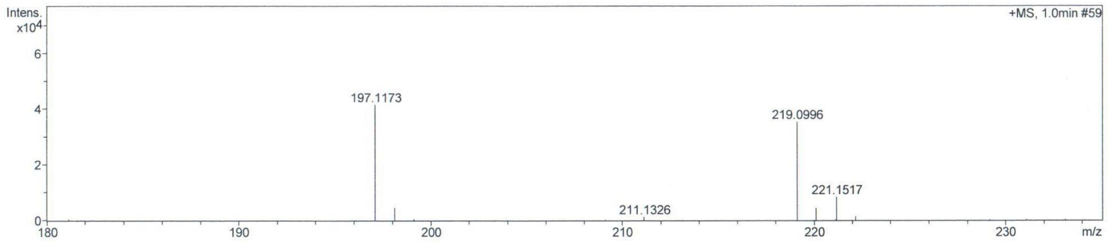

Figure S29. ^{1}H NMR (700 MHz, methanol-d_4) spectrum of minipyrone (**10**).

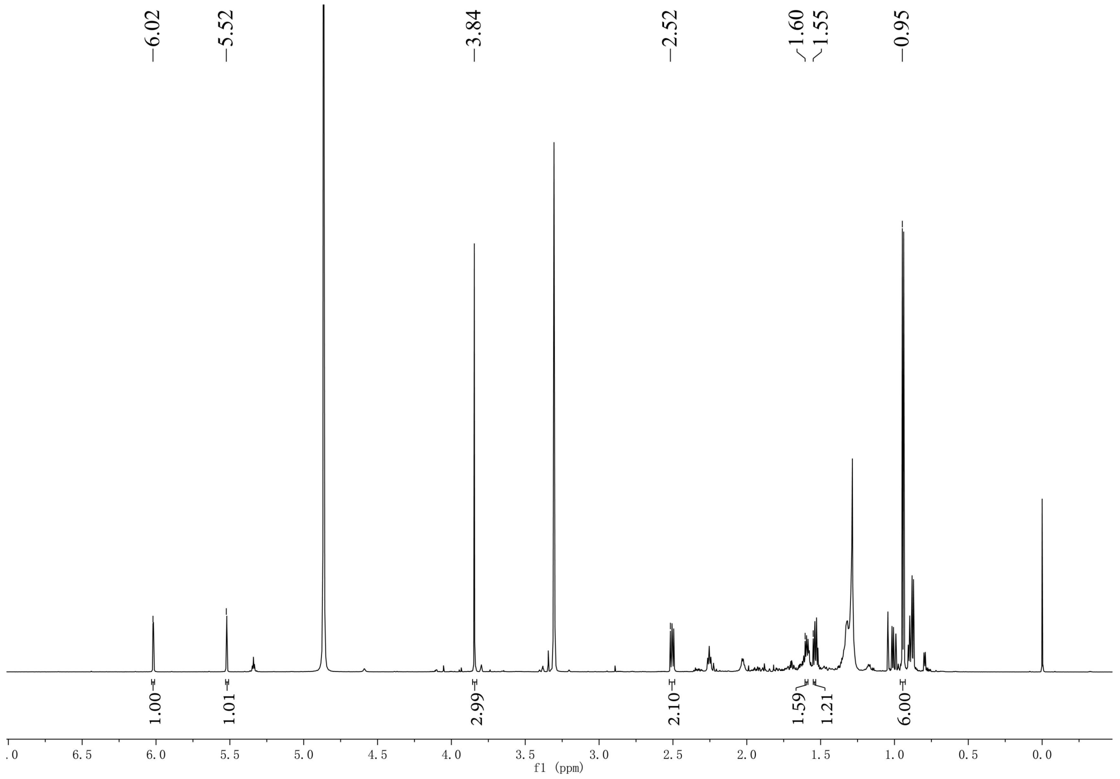

Figure S30. ^{13}C NMR (175MHz, methanol-d_4) spectrum of minipyrone (**10**).

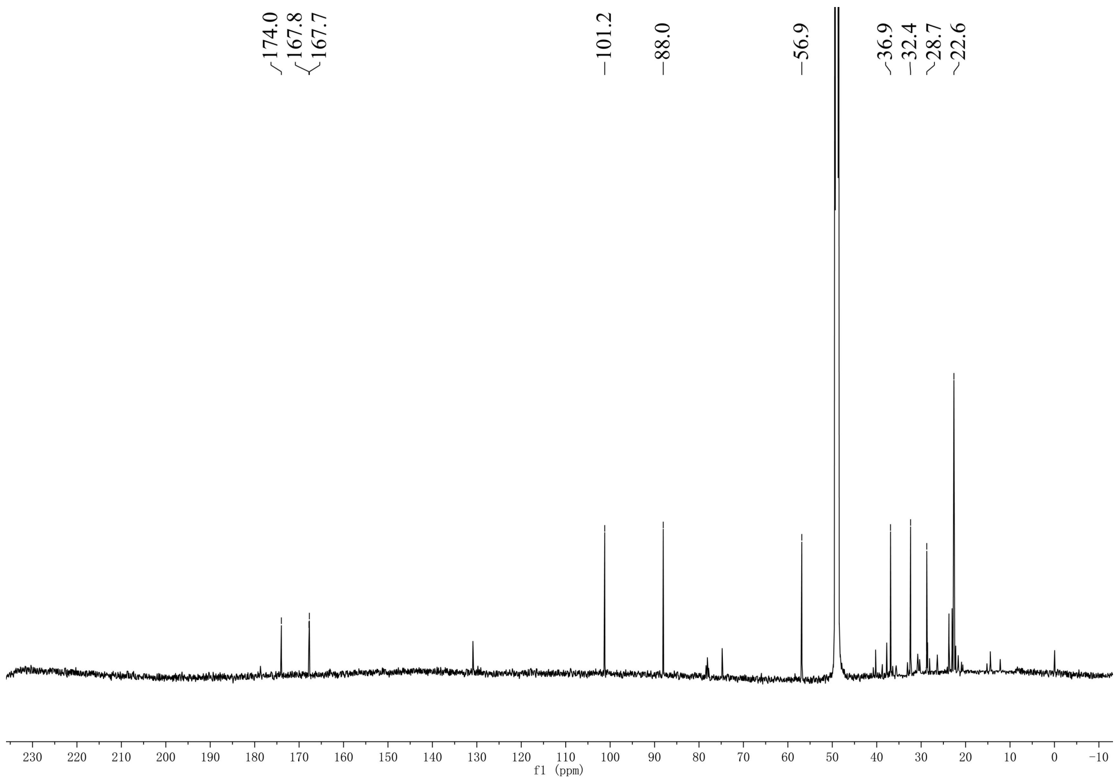

Figure S31. ^{13}C-DEPT NMR (175 MHz, methanol-d_4) spectrum of minipyrone (**10**).

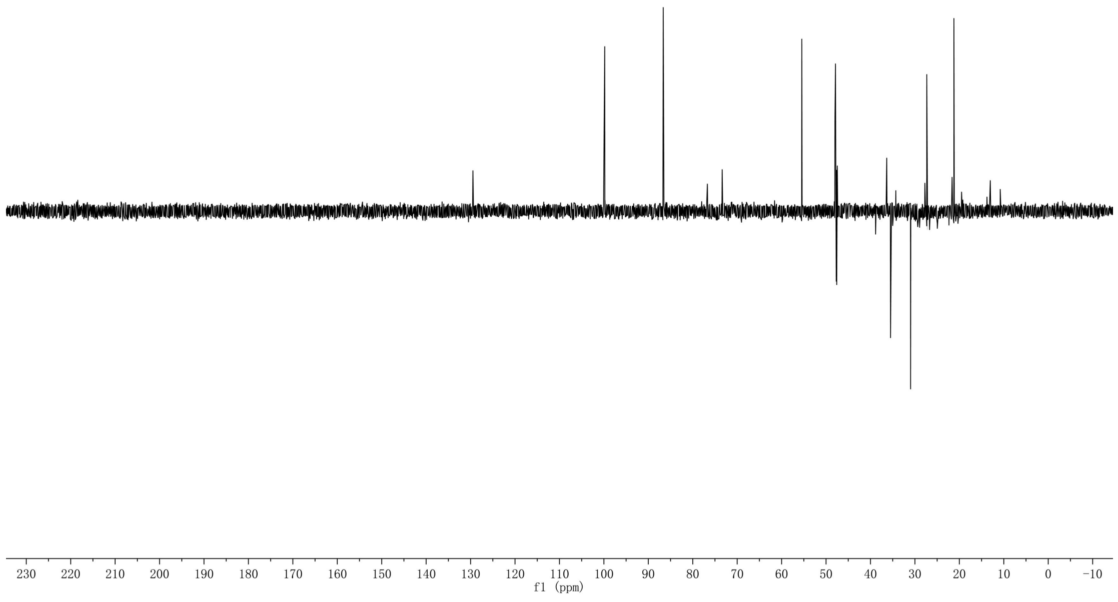

Figure S32. ^{1}H–^{1}H COSY spectrum of minipyrone (**10**).

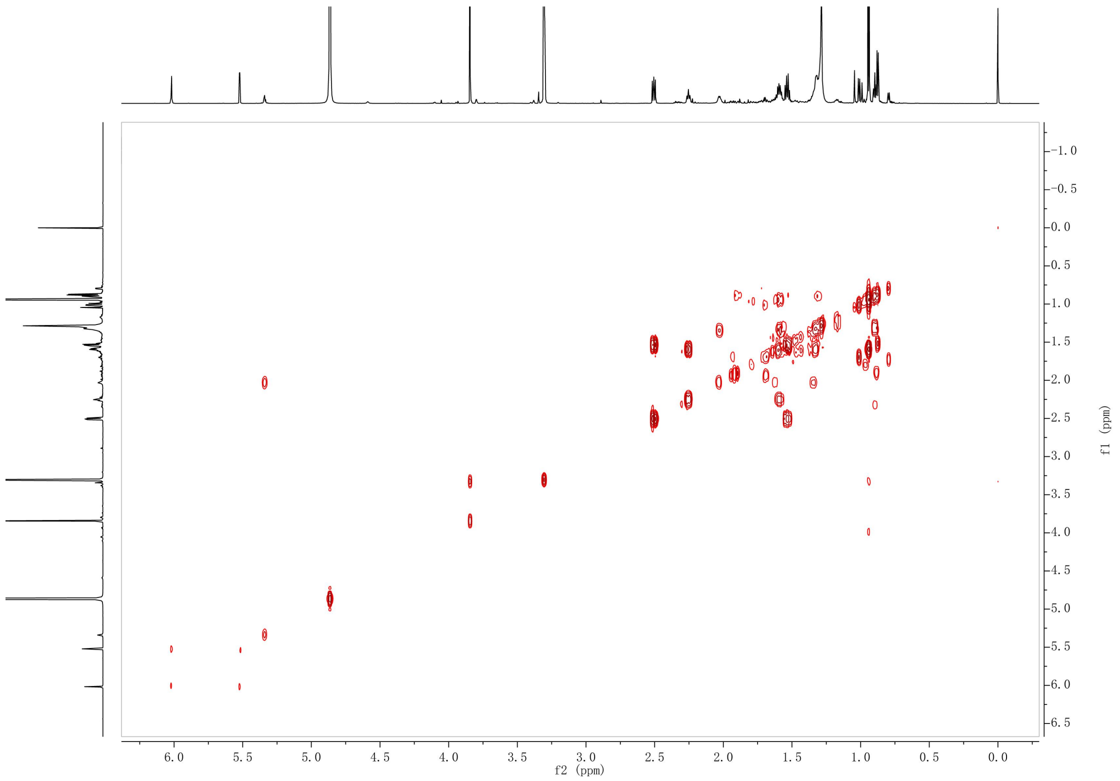

Figure S33. HSQC spectrum of minipyrone (**10**).

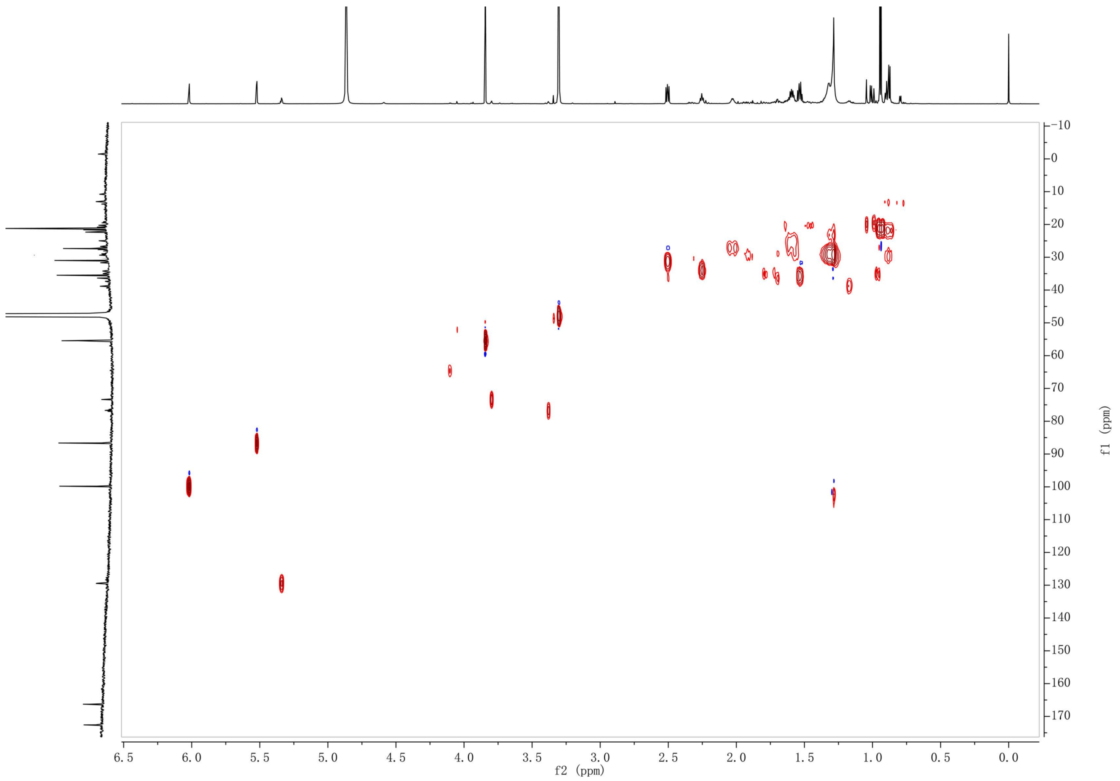

Figure S34. HMBC spectrum of minipyrone (**10**).

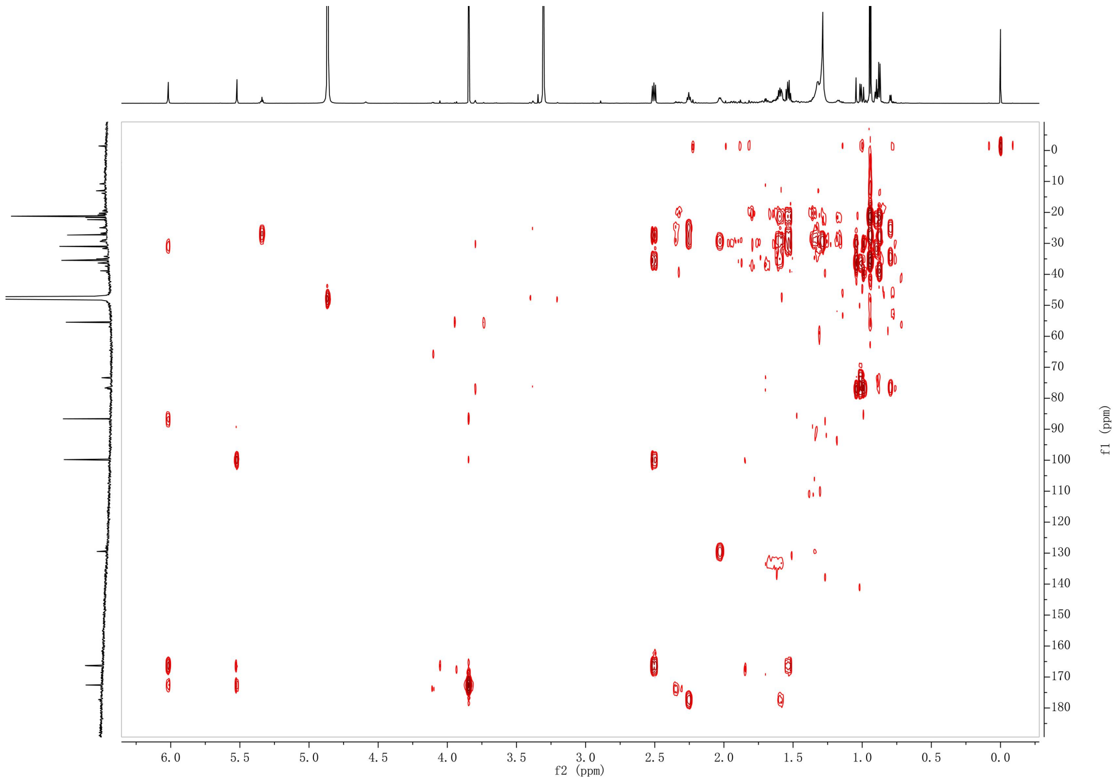

Figure S35. NOESY spectrum of minipyrone (**10**).

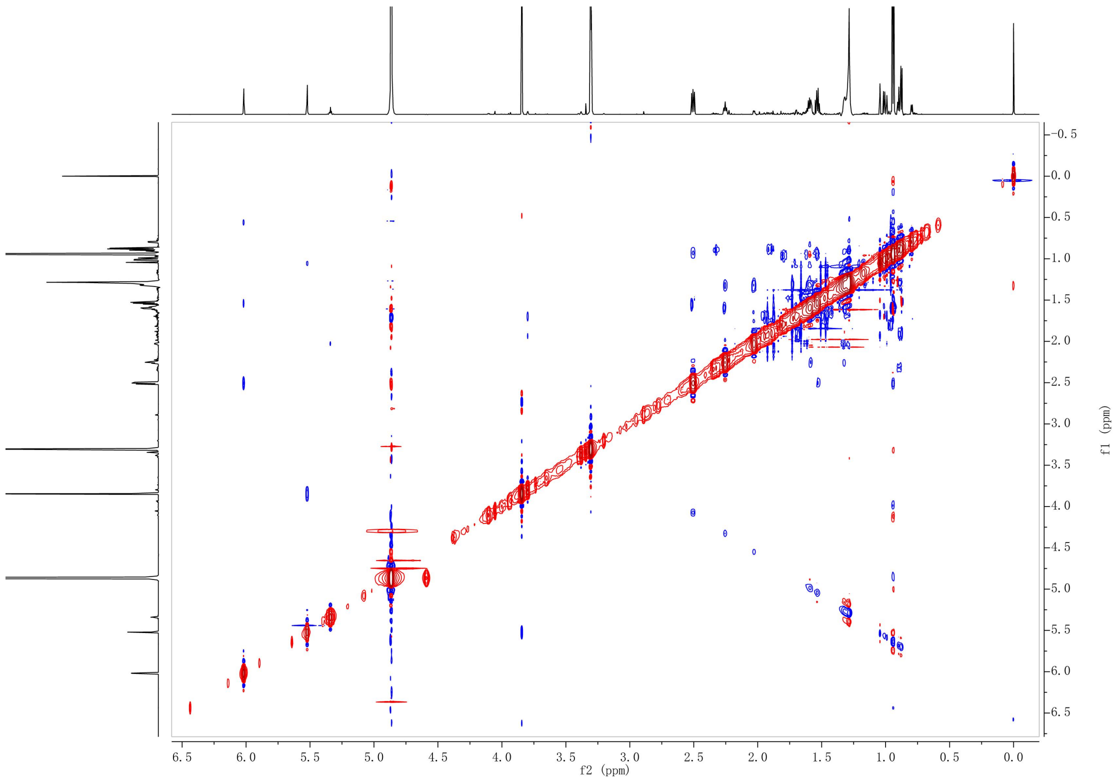

Figure S36. The HPLC-DAD and HRESIMS data of crude extract from *Streptomyces* sp. SCSIO ZS0520.

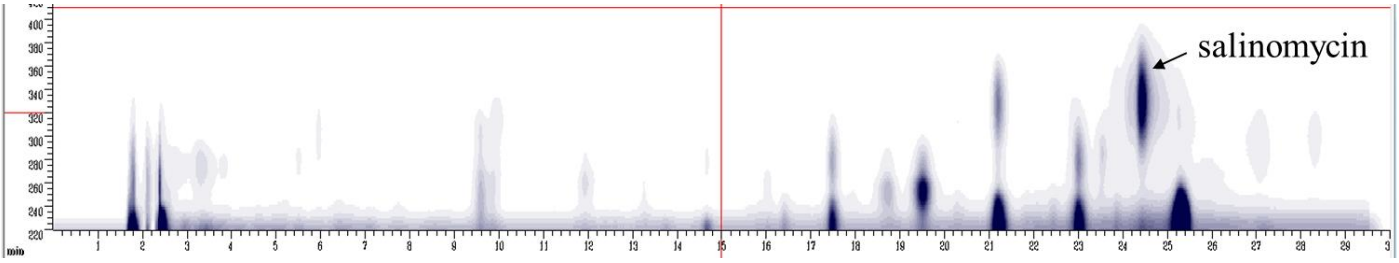

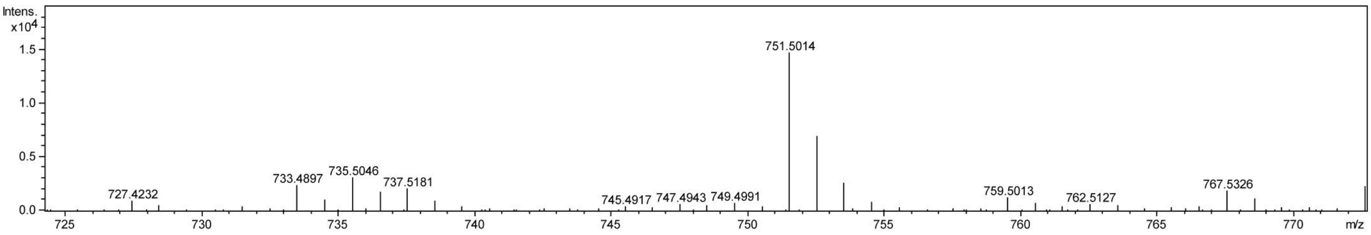

Meas. m/z	#	Formula	Score	m/z	err [mDa]	err [ppm]	mSigma	rdb	e⁻ Conf	N-Rule
751.5014	1	C 42 H 71 O 11	100.00	751.4991	-2.3	-3.0	30.0	7.5	even	ok
